# Cannabidiol Modulates Neuroinflammatory and Estrogen-Related Pathways in a Sex-Specific Manner in a Chronic Stress Model of Depression

**DOI:** 10.3390/cells14020099

**Published:** 2025-01-10

**Authors:** Uri Bright, Irit Akirav

**Affiliations:** 1 Department of Psychology, School of Psychological Sciences, University of Haifa, Haifa 3498838, Israel; uri.bright@yale.edu; 2 The Integrated Brain and Behavior Research Center (IBBRC), University of Haifa, Haifa 3498838, Israel

**Keywords:** UCMS, cannabidiol, endocannabinoids, neuroinflammation, depression

## Abstract

Evidence indicates a bidirectional link between depressive symptoms and neuroinflammation. This study evaluated chronic cannabidiol (CBD) treatment effects in male and female rats subjected to the unpredictable chronic mild stress (UCMS) model of depression. We analyzed the gene expression related to neuroinflammation, cannabinoid signaling, estrogen receptors, and specific microRNAs in the ventromedial prefrontal cortex (vmPFC), CA1, and ventral subiculum (VS). UCMS influenced immobility in a sex-specific manner, increasing it in males and decreasing it in females, effects that were reversed by CBD. CBD also normalized the UCMS-induced upregulation of tumor necrosis factor α (TNF-α) in the CA1 and VS in males. In both sexes, UCMS induced the upregulation of the nuclear factor kappa B subunit 1 (NF-κB1) gene in the VS, which was unaffected by CBD. Additionally, CBD reversed CB1 downregulation in the VS of males but not in the vmPFC of either sex. In males, CBD restored the UCMS-induced downregulation of VS estrogen receptor genes ERα and ERβ. UCMS also altered miR-146a-5p expression, downregulating it in females (VS) and upregulating it in males (CA1), with no CBD effect. These findings highlight the sex-specific mechanisms of CBD’s antidepressant effect, with hippocampal neuroinflammatory and estrogenic pathways playing a key role in males.

## 1. Introduction

Mounting evidence underscores the intricate interplay between depression and inflammation [1], with studies revealing elevated levels of inflammatory markers, such as tumor necrosis factor α (TNF-α) and nuclear factor kappa B subunit 1 (NF-κB1), in individuals with depression [2,3]. Notably, the attenuation of TNF-α and NF-κB1 expression has been associated with improvements in depressive symptoms [4,5].

Cannabidiol (CBD), renowned for its anti-inflammatory and antioxidative properties [6], has emerged as a promising candidate for treating depression [7,8,9,10,11,12,13]. Preclinical studies have demonstrated CBD’s ability to reduce TNF-α expression and NF-κB1 activation [14]. CBD is an inverse agonist of both CB1 and CB2 receptors [15]; it inhibits the enzyme fatty acid amide hydrolase (FAAH), leading to increased levels of anandamide (AEA). Additionally, CBD acts as an agonist of receptors such as transient receptor potential vanilloid 1 (TRPV1), peroxisome proliferator-activated receptor gamma (PPARγ), and serotonin receptor 5-HT1a [16].

Our recent study has highlighted CBD’s efficacy in alleviating depression-like behavior in male rats exposed to unpredictable chronic mild stress (UCMS), a widely used model of depression. These therapeutic effects have been linked to alterations in microRNAs (miR-16 and miR-135) within the ventromedial prefrontal cortex (vmPFC), mediated through the serotonergic 5-HT1a receptor [7].

Several miRNAs, including miR-146, miR-9, and miR-98, have been implicated in stress resilience and depression regulation, influencing inflammatory gene targets [17,18,19,20,21,22,23,24,25,26,27,28,29,30,31]. Elevated miR-146 levels have been associated with TNF-α treatment [23] and correlated with depressive symptoms [17,18,19]. MiR-9 mediated depressive-like symptoms and its downregulation or inhibition decreased depressive-like behavior in UCMS mice, improved the regeneration of hippocampal cells [21], and improved learning and memory in a water maze test [32]. MiR-9 also targets NF-κB1 and inhibits its expression, as was found in studies with immune disease patients [25,26,28]. Mir-98 negatively correlated with depressive symptoms; its expression was lower in the PFC and hippocampus of mice that were subjected to chronic social stress while overexpressing miR-98 led to the alleviation of depressive-like symptoms [22]. MiR-98 also downregulated TNF-α expression [25,29,33], and its inhibition elevated TNF-α [31].

Sex differences in depression prevalence are well-documented, with women exhibiting nearly double the lifetime prevalence compared to men [34]. Preclinical studies suggest that male and female rats may respond differently to stress models and pharmacological treatments [35,36,37], possibly due to hormonal disparities, particularly estrogen; estrogen receptors are abundant both in the brains of males and females, though distributed differently [38]. The estrogen-α (ERα) and estrogen-β (ERβ) receptors play a pivotal role in mediating depressive-like symptoms [39,40], with ERβ implicated in the antidepressant effects of 17β-Estradiol in the forced swimming test (FST) in female rodents [41,42]; intra-hippocampal 17β-Estradiol had a similar antidepressant effect [40].

Our study aimed to elucidate whether the antidepressant properties of CBD are associated with alterations in genes encoding neuroinflammatory, estrogen, and cannabinoid receptors in key brain regions implicated in depression, namely the vmPFC, hippocampal CA1, and ventral subiculum (VS). Additionally, we investigated changes in specific miRNAs associated with neuroinflammation and depression, shedding light on potential mechanisms underlying CBD’s therapeutic effects [19,22,28].

## 2. Materials and Methods

### 2.1. Subjects

Male and female Sprague Dawley rats, 60 days old (Envigo, Jerusalem, Israel) were either housed in groups (Non-UCMS rats) or individually (UCMS rats) in a temperature-controlled environment maintained at 22 ± 2 °C with a 12 h light/dark cycle (lights on at 07:00). The rats were given unrestricted access to water and standard laboratory rodent chow, except during periods specified by the UCMS protocol. The number of animals per group in the behavioral experiments was 10, and in the PCR experiments, it ranged between 8 and 10. Group sizes were determined based on our previous research [7].

### 2.2. UCMS Protocol

The rats underwent a four-week protocol involving mild stressors, delivered in a randomized sequence as previously described in our lab [7] (see Figure S1 and Supplementary Methods). Rats in the non-stressed group were handled but were not subjected to the stress procedure. For elaborated information on the procedure, see Supplementary Materials.

### 2.3. Pharmacological Agents

In the last two weeks of the four-week UCMS protocol, both non-stressed and UCMS-exposed rats were given daily intraperitoneal (i.p.) injections of either vehicle or CBD (Symrise, Holzminden, Germany) (10 mg/kg). The drug solutions, prepared fresh and administered at a volume of 1 mL/kg, were injected between 10:00 and 12:00 pm, regardless of the stress schedule. CBD was dissolved in a mixture of 2% Tween-80 and 98% saline, with dosing based on previous studies conducted in our lab and other research [7,8].

### 2.4. Behavioral Tests

#### 2.4.1. Open Field Test (OFT)

Locomotion was measured in an open-field test. The arena was a 50 × 50 cm open black box, placed in a room lit with red lighting. The box was cleaned between each trial. The rat’s movements were recorded and analyzed with Ethovision (Ethovision ×T 14.0, Noldus Information Technology, NBT Ltd., Jerusalem, Israel) to measure motor activity over a 30 min period.

#### 2.4.2. Forced Swim Test (FST)

The test was performed in a cylindrical water tank (62 cm diameter, 40 cm height) filled with water maintained at a constant temperature of 22 °C. The tank was situated in a room illuminated by red light. The water level was set so that the rat’s hind paws could not touch the bottom. On the first day, rats were exposed to the swim tank for 15 min, and for 5 min on the second day. Video recordings from the second day of each FST session were analyzed by a trained, blinded observer, who assessed the rat’s coping behavior, distinguishing between passive coping (immobility) and active coping (climbing and swimming) strategies.

### 2.5. Weight

Rats’ weights were measured weekly (Table S1).

### 2.6. Quantitative Real-Time PCR (qRT-PCR)

Rats were euthanized, and their brain tissues from the ventromedial prefrontal cortex (vmPFC), hippocampal CA1 region, and ventral subiculum (VS) were harvested using 0.5–1.0 mm punches with a cryostat for biochemical analysis (see Figure S2). RNA was extracted and cDNA was prepared followed by quantitative real-time PCR (qRT-PCR) as described previously [7,43,44] to assess the expression of specific miRNAs (miR-9-5p, miR-98-5p, and miR-146a-5p) and mRNAs (*cnr1*, *cnr2*, *tnf*, *nfkb1*, *esr1*, and *esr2* genes coding to CB1r, CB2r, TNF-α, NF-κB1, Erα, and ERβ, respectively) (mRNA primer sequences are listed in Table S2). A total of 500 ng of RNA was polyadenylated and converted into cDNA using the qScript miRNA cDNA Synthesis Kit (Quanta Biosciences, Gaithersburg, MD, USA). Real-Time SYBR Green qRT-PCR amplification was performed with specific primers (Quanta Biosciences, Gaithersburg, MD, USA) according to the manufacturer’s guidelines. The RT reactions were carried out on a Step One Real-Time PCR system (Applied Biosystems, Waltham, MA, USA). Fold-changes in gene expression were calculated using the ddCt method, withRNU6 used as a reference gene for miRNA and HPRT for mRNA. Some samples were excluded during RNA extraction due to low quality, resulting in a reduced number of samples. As a result, between eight and ten samples were available for each brain region.

### 2.7. Statistical Analyses

The data are presented as means ± SEM. Statistical analysis was performed using three-way ANOVA, two-way ANOVA, and Pearson’s bivariate correlation, as appropriate. Post hoc comparisons were conducted with Tukey’s range test. Significance was defined as *p* ≤ 0.05. Data analysis was carried out using SPSS 27 (IBM, Chicago, IL, USA). The normality assumption was examined using the Kolmogorov–Smirnov and Shapiro–Wilk tests.

## 3. Results

### 3.1. The Effects of Chronic CBD Administration During UCMS on Depressive-Like Symptoms

**Figure 1:**
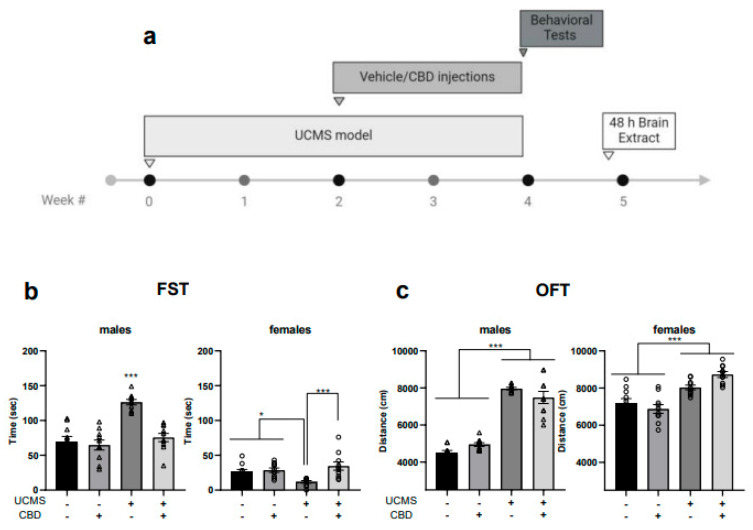
(**a**) Experimental design: Male and female rats were exposed to the UCMS model for four weeks or were not exposed. Non-UCMS and UCMS-exposed rats received daily injections (i.p.) of vehicle or CBD (10 mg/kg) during the last two weeks of the 4-week UCMS model. Behavioral tests, including FST and OFT, followed this. On day 35, rats were euthanized and examined for the expression of the *cnr1*, *cnr2*, *tnf*, *nfkb1*, *esr1*, and *esr2* genes (which encode for CB1, CB2, TNF-α, NF-κB1, ER-α, and ER-β, respectively) and miRNAs (miR-9-5p, miR-98-5p, and miR-146a-5p) in the vmPFC, CA1, and VS. All analyses were conducted using two-way ANOVA [stress × drug (2 × 2); *n* = 8–10 of each sex in each group]. (**b**) Males: UCMS rats treated with a vehicle spent more time immobile than all groups in the FST. Females: UCMS rats treated with a vehicle spent less time immobile than all groups. (**c**): Males and Females: UCMS rats traveled more distance than non-UCMS rats in the OFT. CBD: cannabidiol; FST: forced swim test; miRNAs, miRs: microRNAs; OFT: open field test; UCMS: unpredictable chronic mild stress; vmPFC: ventromedial prefrontal cortex; VS: ventral subiculum *, *p* < 0.05, ***, *p* < 0.001. Each group consisted of 10 rats.

We used a 2 × 2 × 2 design with the main factors being sex, stress (non-UCMS/UCMS), and drug (vehicle/CBD) (see Figure 1a for experimental design). In cases of a significant sex effect or three-way interaction, data from male and female rats were analyzed separately. See Table S3 for detailed analyses of three-way and two-way ANOVA.

In the FST (Figure 1b), univariate ANOVA [sex × stress × drug (2 × 2 × 2)] revealed significant effects of the sex, drug, and stress factors and the interactions stress × sex, sex × drug, and sex × stress × drug on immobility. No significant effects of the stress × drug interactions were found. Two-way ANOVA [stress × drug (2 × 2)] revealed the significant effects of the drug factor (males: F(1,39) = 19.412, *p* < 0.001; females: F(1,39) = 10.602, *p* < 0.01), the stress factor (males: F(1,39) = 28.714, *p* < 0.001), and stress × drug interactions (males: F(1,39) = 13.394, *p* < 0.01; females: F(1,39) = 7.199, *p* < 0.05). This suggests that in males, CBD restored the UCMS-induced increase in immobility, while in females, CBD restored the UCMS-induced decrease in immobility.

Significant effects of the stress and drug factors, and stress × drug interactions on swimming were found in males and of drug × stress in females. This suggests that in males, UCMS decreased swimming time and CBD prevented this effect. No effects on climbing were found (data available in Table S3).

In the OFT (Figure 1c), univariate ANOVA revealed significant effects on locomotion of the sex and stress factors and the following interactions: sex × stress and sex × stress × drug. Two-way ANOVA revealed significant effects of stress (males: F(1,39) = 242.585, *p* < 0.001; females: F(1,39) = 46.286, *p* < 0.001) and stress × drug interactions (males: F(1,39) = 6.143, *p* < 0.05; females: F(1,39) = 6.376, *p* < 0.05), with no effect regarding the drug factor (males: F(1,39) = 0.001, ns; females: F(1,39) = 0.943, ns), suggesting that UCMS led to increased locomotion in both sexes, regardless of CBD treatment. Also, we found no significant effects of stress or CBD on the time spent in the center of the arena during the first 5 min of the test in males and females (see Table S3), suggesting that UCMS did not cause anxiety-like behavior.

### 3.2. The Effects of Chronic CBD Administration During UCMS on Cannabinoid Receptors, Inflammatory Markers, and Estrogen Receptor Gene Expression

#### 3.2.1. *cnr1*

**Figure 2:**
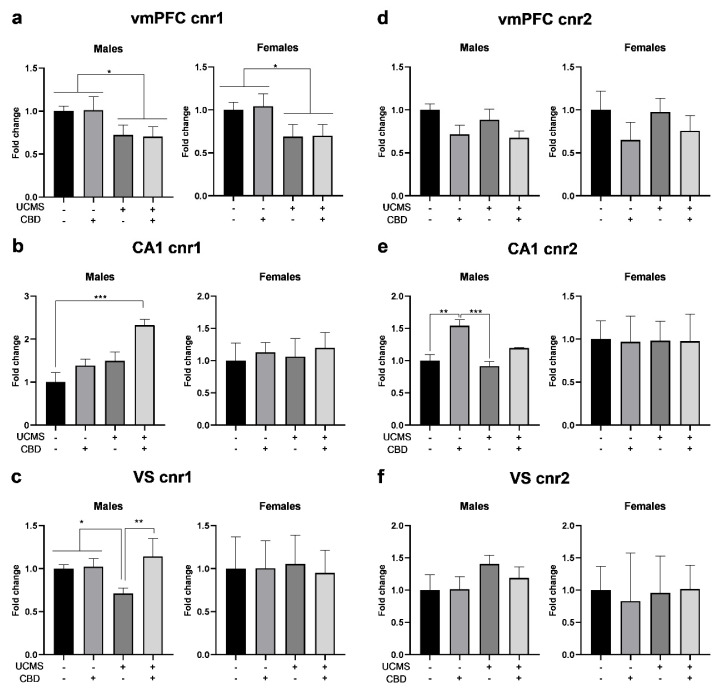
The effects of CBD treatment on CB1r and CB2r mRNA (*cnr1* and *cnr2*, respectively) expression in the vmPFC, CA1, and VS in rats exposed to UCMS. (**a**) Males and Females: UCMS downregulated *cnr1* levels in the vmPFC. (**b**) Males: UCMS rats treated with CBD demonstrated *cnr1* upregulation in the CA1 compared to non-UCMS rats treated with a vehicle. Females: there were no differences between the groups. (**c**) Males: UCMS rats treated with a vehicle demonstrated downregulated *cnr1* levels in the VS compared to all groups. Females: there were no differences between the groups. (**d**) Males and females: CBD downregulated *cnr2* in the vmPFC. (**e**) Males: non-UCMS rats treated with CBD demonstrated upregulated *cnr1* levels in the CA1 compared to non-UCMS and UCMS rats treated with vehicle. Females: there were no differences between the groups. (**f**) Males and Females: there were no differences between the groups in cnr2 in the VS. CBD: cannabidiol; UCMS: unpredictable chronic mild stress; vmPFC: ventromedial prefrontal cortex; VS: ventral subiculum. *, *p* < 0.05; **, *p* < 0.01; ***, *p* < 0.001 indicate statistically significant effects followed by post hoc comparisons. There were 8–10 samples per group.

In the vmPFC (Figure 2a), univariate ANOVA [sex × stress × drug (2 × 2 × 2)] revealed significant effects of the stress factor but not of the drug factor or any of the interactions. Two-way ANOVA [stress × drug (2 × 2)] revealed significant effects of stress (males: F(1,35) = 17.396, *p* < 0.001; females: F(1,34) = 17.912, *p* < 0.001) but not of the drug factor (males: F(1,35) = 0.011, ns; females: F(1,34) = 0.098, ns) or stress × drug interactions (males: F(1,35) = 0.072, ns; females: F(1,34) = 0.019, ns), suggesting that UCMS led to the downregulation of *cnr1* regardless of CBD treatment.

In the CA1 (Figure 2b), univariate ANOVA revealed significant effects of the sex, stress, and drug factors. Two-way ANOVA revealed significant effects of the drug factor (F(1,36) = 9.757, *p* < 0.01) and the stress factor (F(1,36) = 13.617, *p* < 0.001) in males but not in females (drug: F(1,37) = 0.493, ns; stress: F(1,37) = 0.124, ns). This suggests that CBD treatment to UCMS males resulted in the upregulation of *cnr1*. The stress × drug interaction was not significant in males (F(1,36) = 0.019, ns) or females (F(1,37) = 0.001, ns).

In the VS (Figure 2c), univariate ANOVA revealed the significant effects of the sex factor. Two-way ANOVA revealed the significant effects of the drug factor (F(1,30) = 8.394, *p* < 0.01) and the stress × drug interaction (F(1,30) = 6.915, *p* < 0.05) in males but not in females (drug: F(1,28) = 0.001, ns; stress × drug: F(1,28) = 0.001, ns). This suggests that CBD prevented *cnr1* downregulation in male UCMS rats. Stress was not significant in males (F(1,30) = 2.708, ns) or females (F(1,28) = 0.066, ns).

#### 3.2.2. *cnr2*

In the vmPFC (Figure 2d), univariate ANOVA (2 × 2 × 2) revealed the significant effects of the sex and drug factors but not of the stress factor or any of the interactions. Two-way ANOVA (2 × 2) revealed the significant effects of the drug factor (males: F(1,34) = 6.927, *p* < 0.05; females: F(1,32) = 6.706, *p* < 0.05) but not of stress (males: F(1,34) = 0.614, ns; females: F(1,32) = 0.233, ns) or stress × drug interaction (males: F(1,34) = 0.081, ns; females: F(1,32) = 0.454, ns), suggesting that CBD downregulated *cnr2* in both sexes irrespective of UCMS.

In the CA1 (Figure 2e), univariate ANOVA revealed the significant effects of the sex factor but not of the stress or drug factors, or any of the interactions. Two-way ANOVA revealed the significant effects of the drug (F(1,32) = 22.341, *p* < 0.001) and stress factors (F(1,32) = 0.5.439, *p* < 0.05) in males, but not in females: (drug: F(1,34) = 0.110, ns; stress: F(1,34) = 0.001, ns), suggesting that CBD treatment led to the upregulation of *cnr2* in males. Stress × drug interaction was not significant in males (F(1,32) = 1.302, ns) or females (F(1,34) = 0.004, ns).

In the VS (Figure 2f), univariate ANOVA revealed the significant effects of the sex factor but not of the stress or drug factors or any of the interactions. Two-way ANOVA revealed no significant effects of the drug factor (males: F(1,30) = 0.208, ns; females: F(1,29) = 0.024, ns), the stress factor (males: F(1,30) = 2.174, ns; females: F(1,29) = 0.039, ns), or stress × drug interaction (males: F(1,30) = 0.290, ns; females: F(1,29) = 0.093, ns), suggesting that in both sexes, neither UCMS nor CBD affected *cnr2* expression.

#### 3.2.3. *tnf*

**Figure 3:**
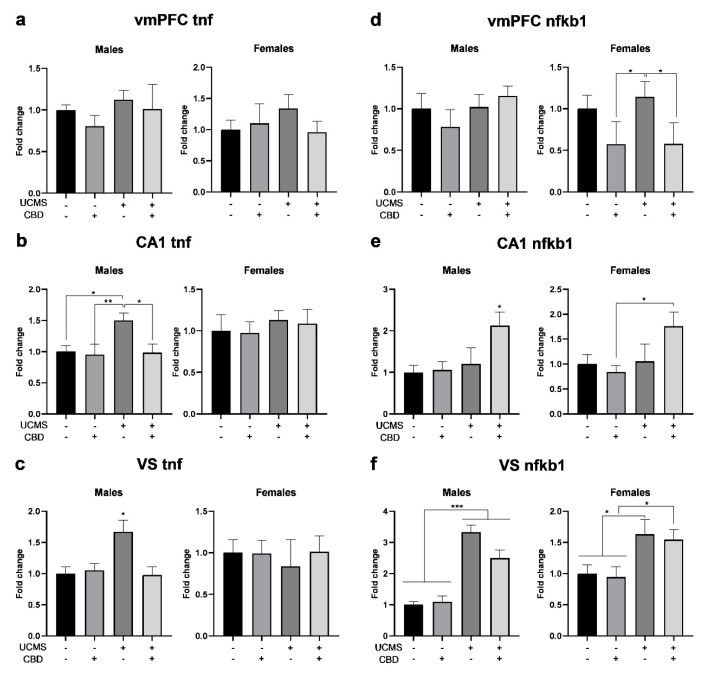
The effects of CBD treatment on the TNF-α and NF-κB1 mRNA (*tnf* and *nfkb1*, respectively) expression in the vmPFC, CA1, and VS in rats exposed to UCMS. (**a**) Males and Females: there were no differences between the groups in *tnf* in the vmPFC. (**b**) Males: UCMS rats treated with vehicle demonstrated *tnf* upregulation in the CA1 compared to all groups. Females: there were no differences between the groups. (**c**) Males: UCMS rats treated with vehicle demonstrated *tnf* upregulation in the VS compared to all groups. Females: there were no differences between the groups. (**d**) Males: there were no differences between the groups in *nfkb1* in the vmPFC. Females: UCMS rats treated with vehicle showed higher *nfkb1* expression compared to non-UCMS and UCMS rats treated with CBD. (**e**) Males: UCMS rats treated with CBD demonstrated upregulated *nfkb1* levels in the CA1 compared to all groups. Females: UCMS rats treated with CBD showed higher *nfkb1* expression than non-UCMS rats treated with CBD. (**f**) Males: UCMS rats demonstrated *nfkb1* upregulation in the VS compared to non-UCMS rats. Females: UCMS rats treated with vehicle showed higher *nkfb1* expression in the VS than the non-UCMS groups. UCMS rats treated with CBD showed higher *nfkb1* expression than non-UCMS rats treated with CBD. CBD: cannabidiol; UCMS: unpredictable chronic mild stress; vmPFC: ventromedial prefrontal cortex; VS: ventral subiculum. *, *p* < 0.05, **, *p* < 0.01, ***, *p* < 0.001. There were 8–10 samples per group.

In the vmPFC (Figure 3a), univariate ANOVA (2 × 2 × 2) revealed the significant effects of the sex factor but not of the stress or drug factors or any of the interactions. Two-way ANOVA (2 × 2) revealed no significant effect of the drug factor (males: F(1,36) = 1.060, ns; females: F(1,33) = 0.576, ns), the stress factor (males: F(1,36) = 1.211, ns; females: F(1,33) = 0.243, ns), or the stress × drug interaction (males: F(1,36) = 0.122, ns; females: F(1,33) = 1.894, ns).

In the CA1 (Figure 3b), univariate ANOVA revealed the significant effects of the sex and stress factors but not of the drug factor or any of the interactions. Two-way ANOVA revealed the significant effects of the drug factor (F(1,36) = 6.488, *p* < 0.05), the stress factor (F(1,36) = 5.668 *p* < 0.05), and the stress × drug interaction (F(1,36) = 4.085, *p* < 0.05) in males, but not in females (drug: F(1,36) = 0.086, ns; stress: F(1,36) = 1.097, ns; stress × drug interaction: F(1,36) = 0.003, ns), suggesting that CBD prevented the UCMS-induced upregulation of *tnf* in males.

In the VS (Figure 3c), univariate ANOVA revealed significant effects of the sex factor and the sex × stress × drug interaction, but not of the stress or drug factors or other interactions. Two-way ANOVA revealed the significant effects of the drug factor (F(1,29) = 4.592, *p* < 0.05) and stress × drug interaction (F(1,29) = 6.718, *p* < 0.05) in males but not in females (drug: F(1,28) = 0.388, ns; sex × drug: F(1,28) = 0.462, ns), suggesting that CBD prevented the upregulation of *tnf* in male UCMS rats. Stress was not significant in males (F(1,29) = 3.704, ns) or females (F(1,28) = 0.290, ns).

#### 3.2.4. *nfkb1*

In the vmPFC (Figure 3d), univariate ANOVA (2 × 2 × 2) revealed the significant effects of the sex factor and the sex × drug interaction, but not of the stress or drug factors or other interactions. Two-way ANOVA (2 × 2) revealed the significant effects of the drug factor in females (F(1,34) = 14.913, *p* < 0.001) but not in males (F(1,32) = 0.138, ns), with no effects of stress (males: F(1,32) = 1.565, ns; females: F(1,34) = 0.195, ns) or the stress × drug interaction (males: F(1,32) = 1.264, ns; females: F(1,34) = 0.157, ns), suggesting that in females, CBD resulted in the downregulation of *nfkb1*, irrespective of UCMS.

In the CA1 (Figure 3e), univariate ANOVA revealed the significant effects of the sex, stress, and drug factors and the stress × drug interaction. Two-way ANOVA revealed the significant effects of stress (males: F(1,36) = 4.865, *p* < 0.05; females: F(1,35) = 5.283, *p* < 0.05). The drug factor was significant in males (F(1,36) = 4.735, *p* < 0.05) but not in females (F(1,35) = 0.966, ns). The stress × drug interaction was not significant in males (F(1,36) = 3.419, ns) or females (F(1,35) = 3.953, ns).

In the VS (Figure 3f), univariate ANOVA revealed the significant effects of the sex and stress factors and the sex × stress interaction, but not of the drug factor or other interactions. Two-way ANOVA revealed the significant effects of stress (males: F(1,31) = 44.963, *p* < 0.001; females: F(1,27) = 15.462, *p* < 0.001) but not of the drug factor (males: F(1,31) = 0.430, ns; females: F(1,27) = 0.214, ns) and the stress × drug interaction (males: F(1,31) = 1.547, ns; females: F(1,27) = 0.001, ns), suggesting that in both sexes, UCMS led to the upregulation of *nfkb1*, regardless of CBD treatment.

#### 3.2.5. *esr1*

**Figure 4:**
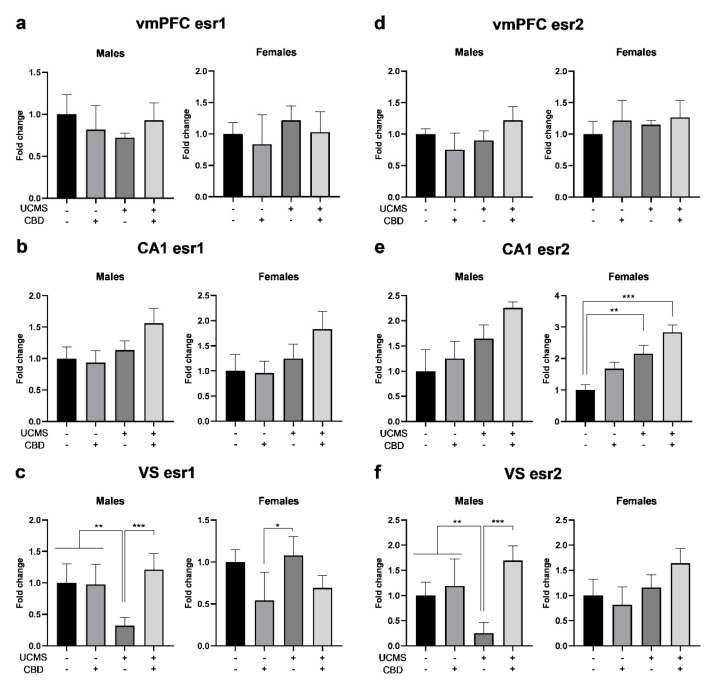
The effects of CBD treatment on ERα and ERβ mRNA (*esr1* and *esr2*, respectively) expression in the vmPFC, CA1, and VS in rats exposed to UCMS. (**a**) Males and remales: there were no differences between the groups in *esr1* in the vmPFC. (**b**) Males and females: UCMS rats demonstrated *esr1* upregulation in the CA1. (**c**) Males: UCMS rats treated with vehicle demonstrated *esr1* downregulation in the VS compared to all groups. Females: UCMS rats treated with vehicle demonstrated higher *esr1* expression than non-UCMS-CBD rats. (**d**) Males and females: there were no differences between the groups in *esr2* in the vmPFC. (**e**) Males: UCMS upregulated *esr2* in the CA1. Females: UCMS rats demonstrated *esr2* upregulation compared to non-UCMS rats treated with a vehicle. (**f**) Males: UCMS rats treated with vehicle demonstrated *esr2* downregulation in the VS compared to all groups. Females: there were no differences between the groups. CBD: cannabidiol; UCMS: unpredictable chronic mild stress; vmPFC: ventromedial prefrontal cortex; VS: ventral subiculum. *, *p* < 0.05; **, *p* < 0.01; ***, *p* < 0.001 indicate statistically significant effects followed by post hoc comparisons. There were 8–10 samples per group.

In the vmPFC (Figure 4a), univariate ANOVA (2 × 2 × 2) revealed no significant effect of the sex, stress, or drug factors or any of the interactions.

Two-way ANOVA (2 × 2) revealed no significant effect of the drug factor (males: F(1,35) = 0.031, ns; females: F(1,34) = 0.561, ns) the stress factor (males: F(1,35) = 0.503, ns; females: F(1,34) = 0.762, ns), or stress × drug interaction (male: F(1,35) = 2.5601, ns; females: F(1,34) = 0.001, ns).

In the CA1 (Figure 4b), univariate ANOVA revealed the significant effects of the sex and stress factors but not of the drug factor or any of the interactions. Two-way ANOVA revealed the significant effects of stress (males: F(1,35) = 5.650, *p* < 0.05; females: F(1,36) = 4.462, *p* < 0.05) but not the drug factor (males: F(1,35) = 0.903, ns; females: F(1,36) = 0.270, ns) or the stress × drug interaction (males: F(1,35) = 2.064, ns; females: F(1,36) = 1.744, ns), suggesting that in both sexes, UCMS led to the upregulation of *esr1* regardless of CBD treatment.

In the VS (Figure 4c), univariate ANOVA revealed the significant effects of the following interactions: sex × stress, sex × drug, stress × drug, and sex × stress × drug, but not of the sex, stress, or drug factors. Two-way ANOVA revealed the significant effects of the drug factor in males (F(1,31) = 12.044, *p* < 0.01) and females (F(1,31) = 10.732, *p* < 0.01). Stress (F(1,31) = 6.010, *p* < 0.05) and stress × drug interactions (F(1,31) = 13.030, *p* < 0.01) were significant in males but not in females (stress: F(1,31) = 0.962, ns; stress × drug: F(1,31) = 0.275, ns), suggesting that in males, CBD prevented the UCMS-induced downregulation of *esr1*.

#### 3.2.6. *esr2*

In the vmPFC (Figure 4d), univariate ANOVA (2 × 2 × 2) revealed the significant effects of the sex factor but not of the stress or drug factors or any of the interactions. Two-way ANOVA (2 × 2) revealed significant effects of stress × drug interaction (F(1,35) = 4.821, *p* < 0.05) in males but not in females (F(1,35) = 0.088, ns). No significant effect was found for the drug factor (males: F(1,35) = 0.004, ns; females: F(1,35) = 0.755, ns) or the stress factor (males: F(1,35) = 2.008, ns; females: F(1,35) = 0.280, ns).

In the CA1 (Figure 4e), univariate ANOVA revealed the significant effects of the sex, stress, and drug factors, but not any of the interactions. Two-way ANOVA revealed the significant effects of stress (males: F(1,35) = 6.485, *p* < 0.05; females: F(1,36) = 17.306, *p* < 0.001). The drug factor was significant in females (F(1,36) = 6.483, *p* < 0.05) but not in males (F(1,35) = 1.351, ns). Stress × drug interactions were not significant in males (F(1,35) = 0.023, ns) or females (F(1,36) = 0.611, ns), suggesting that UCMS led to the upregulation of *esr2* in both sexes.

In the VS (Figure 4f), univariate ANOVA revealed the significant effects of the sex and drug factors and the following interactions: sex × stress, sex × drug, and stress × drug. Two-way ANOVA revealed the significant effects of the drug factor (F(1,31) = 17.549, *p* < 0.001), the stress factor (F(1,31) = 4.250, *p* < 0.05) and the stress × drug interaction (F(1,31) = 12.1954, *p* < 0.01) in males, but not in females (drug F(1,31) = 0.115, ns; stress: F(1,31) = 3.955, ns; stress × drug interaction: F(1,31) = 1.687, ns), suggesting that in males, CBD prevented the UCMS-induced downregulation of *esr2*.

The distribution of estrus phases in each group of female rats was observed on the first day of behavioral tests, which coincided with the open field test. A similar distribution of rats across the diestrus, proestrus, and estrus phases was noted within each group (see Table S4 for estrus phase distribution and Table S5 for correlations between estrus phase and behavioral phenotype).

To explore the association between depressive-like behavior and gene expression, Pearson’s bivariate correlation tests were conducted between the behavioral measurements and mRNA expression in the vmPFC, CA1, and VS in males (Supplemental Material, Table S6) and females (Table S7).

### 3.3. The Effects of Chronic CBD Administration During UCMS on miRNA Expression in Male and Female Rats

#### 3.3.1. miR-9-5p

**Figure 5:**
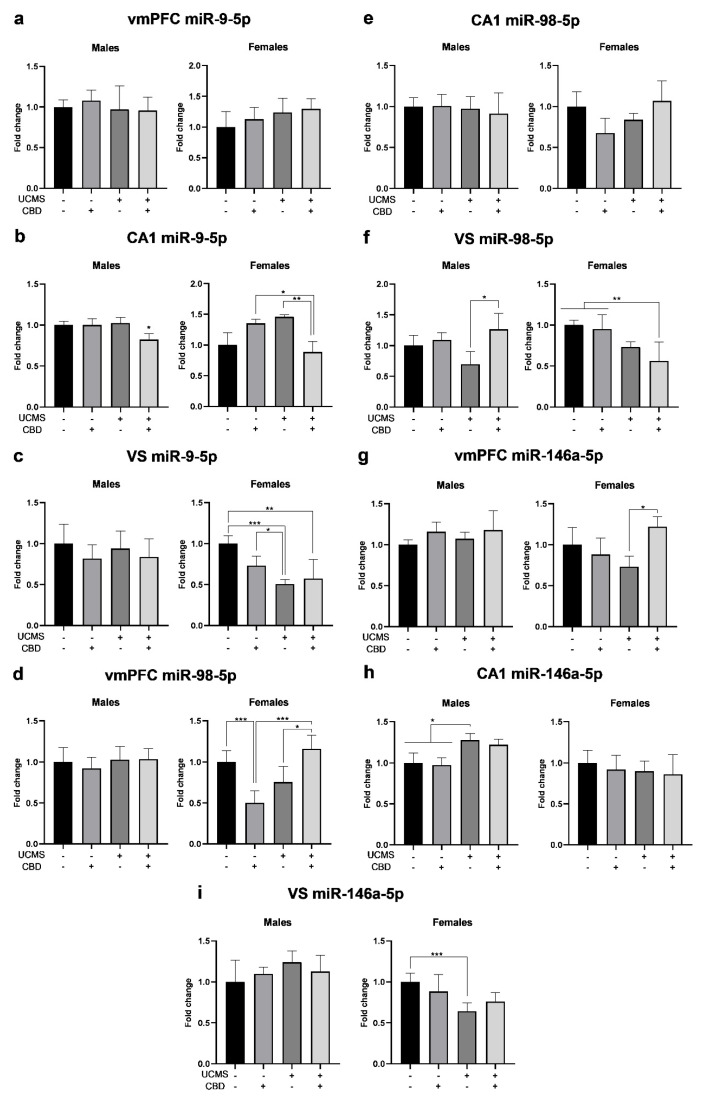
The effects of CBD treatment on miR-9-5p, miR-98-5p, and miR-146a-5p expression in the vmPFC, CA1, and VS in rats exposed to UCMS. (**a**) Males and females: there were no differences between the groups in miR-9-5p in the vmPFC. (**b**) Males: there were no differences between the groups in the CA1. Females: UCMS rats treated with CBD demonstrated lower miR-9-5p expression than UCMS rats treated with vehicle and non-UCMS rats treated with CBD. (**c**) Males: there were no differences between the groups in the VS. Females: UCMS rats treated with a vehicle showed lower miR-9-5p expression than non-UCMS rats. UCMS rats treated with CBD showed lower miR-9-5p expression than non-UCMS rats treated with vehicle. (**d**) Males: there were no differences in miR-98-5p in the vmPFC between the groups. Females: non-UCMS rats treated with CBD showed lower miR-98-5p expression than non-UCMS-Veh rats and UCMS-CBD rats. UCMS rats treated with a vehicle showed lower miR-98-5p expression than UCMS-CBD rats. (**e**) Males and females: there were no differences between the groups in miR-98-5p in the CA1. (**f**) Males: UCMS rats treated with CBD demonstrated the upregulation of miR-98-5p in the VS, while UCMS rats treated with a vehicle demonstrated the downregulation of miR-98-5p. Females: UCMS rats treated with CBD demonstrated lower miR-98-5p expression than non-UCMS rats. (**g**) Males and females: there were no differences between the groups in miR-146a-5p in the vmPFC. (**h**) Males: UCMS rats treated with a vehicle showed the upregulation of miR-146a-5p in the CA1 and were not significantly different from UCMS rats treated with CBD. Females: there were no differences between the groups. (**i**) Males: there were no differences between the groups miR-146a-5p in the VS. Females: UCMS rats treated with a vehicle demonstrated lower miR-146a-5p expression than non-UCMS-Veh rats. CBD: cannabidiol; miR; microRNA; UCMS: unpredictable chronic mild stress; vmPFC: ventromedial prefrontal cortex; VS: ventral subiculum. *, *p* < 0.05, **, *p* < 0.01, ***, *p* < 0.001. There were 8–10 samples per group.

In the vmPFC (Figure 5a), univariate ANOVA (2 × 2 × 2) revealed significant effects of the sex factor but not of the stress factor, the drug factor, or any of the interactions.

Two-way ANOVA (2 × 2) revealed no significant effects of the drug factor (males: F(1,31) = 0.068, ns; females: F(1,33) = 0.335, ns), the stress factor (males: F(1,31) = 0.388, ns; females: F(1,33) = 1.564, ns), or the stress × drug interaction (males: F(1,31) = 0.136, ns; females: F(1,33) = 0.071, ns).

In the CA1 (Figure 5b), univariate ANOVA revealed significant effects for the sex factor and the interactions stress × drug and sex × stress × drug, but not for the stress or drug factors or any of the other interactions. Two-way ANOVA revealed the significant effects of stress × drug interaction (males: F(1,34) = 5.938, *p* < 0.05; females: F(1,34) = 17.087, *p* < 0.001). Significant effects of the drug factor were found in males (F(1,34) = 5.753, *p* < 0.05) but not in females (F(1,34) = 1.026, ns). Stress was not significant in males (F(1,34) = 3.535, ns) or females (F(1,34) = 0.052, ns), suggesting that CBD treatment downregulated miR-9-5p in UCMS rats.

In the VS (Figure 5c), univariate ANOVA revealed the significant effects of the sex and stress factors and sex × stress interaction, but not of the drug factor or any of the other interactions. Two-way ANOVA revealed the significant effects of stress (F(1,34) = 24.911, *p* < 0.001) and stress × drug interaction (F(1,34) = 5.598, *p* < 0.05) in females, but not in males (stress: F(1,28) = 0.016, ns; stress × drug: F(1,28) = 0.091, ns), suggesting that in females, UCMS downregulated miR-9-5p. The drug factor was not significant in males (F(1,28) = 1.137, ns) or females (F(1,34) = 1.045, ns).

#### 3.3.2. miR-98-5p

In the vmPFC (Figure 5d), univariate ANOVA (2 × 2 × 2) revealed the significant effects of the sex and stress factors and the following interactions: stress × drug and sex × stress × drug but not of the drug factor and any of the other interactions. Two-way ANOVA revealed the significant effects of stress (F(1,33) = 6.154, *p* < 0.05) and stress × drug interaction (F(1,33) = 24.91, *p* < 0.001) in females but not in males (stress: F(1,31) = 0.480, ns; stress × drug interaction: F(1,31) = 0.194, ns), suggesting CBD downregulated miR-98-5p in non-stressed females. The drug factor was not significant in both males (F(1,31) = 0.122, ns) and females (F(1,33) = 1.305, ns).

In the CA1 (Figure 5e), univariate ANOVA revealed the significant effects of sex × stress × drug interaction, but not of stress, drug, or any of the other interactions. Two-way ANOVA revealed the significant effects of stress × drug interaction in females (F(1,34) = 6.102, *p* < 0.05) but not in males (F(1,33) = 0.096, ns). The stress and drug factors were not significant in males (stress: F(1,33) = 0.281, ns; drug: F(1,33) = 0.064, ns) or females (stress: F(1,34) = 1.203, ns; drug: F(1,34) = 0.344, ns).

In the VS (Figure 5f), univariate ANOVA revealed the significant effects of the sex and stress factors and the interactions sex × drug and sex × stress × drug, but not of the drug factor and the other interactions. Two-way ANOVA revealed the significant effects of the drug factor in males (F(1,28) = 5.955, *p* < 0.05) but not in females (F(1,34) = 2.378, ns), and the significant effects of stress in females (F(1,34) = 17.302, *p* < 0.001) but not in males (F(1,28) = 0.587, ns), suggesting that CBD led to the upregulation of miR-98-5p in UCMS males, while in females, UCMS led to the downregulation of miR-98-5p. Stress × drug interaction was not significant in males (F(1,28) = 3.296 ns) or females (F(1,34) = 1.097, ns).

#### 3.3.3. miR-146a-5p

In the vmPFC (Figure 5g), univariate ANOVA (2 × 2 × 2) revealed the significant effects of sex and sex × stress × drug interaction, but not of stress, drug, or any of the other interactions.

Two-way ANOVA (2 × 2) revealed the significant effects of stress × drug interaction in females (F(1,33) = 7.823, *p* < 0.01) but not in males (F(1,31) = 0.064, ns). The stress and drug factors were not significant in males (stress: F(1,31) = 0.160, ns; drug: F(1,31) = 1.246, ns) or females (stress: F(1,33) = 0.005, ns; drug: F(1,33) = 2.812, ns).

In the CA1 (Figure 5h), univariate ANOVA revealed the significant effects of the sex factor and sex × stress interaction, but not of the stress or drug factors or any of the other interactions. Two-way ANOVA revealed the significant effects of stress (F(1,34) = 13.529, *p* < 0.001) in males but not in females (F(1,34) = 0.465, ns), suggesting that in males, UCMS led to upregulation of miR-146a-5p. The drug factor and stress × drug interaction were not significant in males (drug: F(1,34) = 0.286 ns; stress x drug: F(1,34) = 0.017, ns) or females (drug: F(1,34) = 0.259, ns; stress × drug: F(1,34) = 0.028, ns).

In the VS (Figure 5i), univariate ANOVA revealed the significant effects of the sex factor and sex × stress interaction, but not of stress, drug, or any of the other interactions. Two-way ANOVA revealed the significant effects of stress (F(1,31) = 9.293, *p* < 0.01) in females but not in males (F(1,28) = 0.888, ns), suggesting that in females, UCMS led to the downregulation of miR-146a-5p. The drug factor and stress × drug interaction was not significant in males (drug: F(1,28) = 0.001, ns; stress × drug: F(1,28) = 0.527, ns) or females (drug: F(1,31) = 0.048, ns; stress × drug: F(1,31) = 2.262, ns).

To explore the association between depressive-like behavior and microRNA expression, Pearson’s bivariate correlation tests were conducted between the behavioral measurements and microRNA expression in the vmPFC, CA1, and VS in males (Supplementary Materials, Table S8) and females (Table S9).

## 4. Discussion

In this study, we investigated the sex-specific effects of chronic CBD treatment on depressive-like behaviors in male and female rats exposed to UCMS. We explored the potential involvement of neuroinflammatory genes, ECS-related genes, estrogen-associated genes, and miRNAs known to modulate depression and neuroinflammation. Our findings highlight the differential impacts of CBD treatment on depressive phenotypes across sexes and elucidate the underlying molecular mechanisms. Specifically, we demonstrate that CBD prevents the impact of UCMS on *cnr1*, *tnf*, *esr1*, and *esr2* expression in the hippocampus of males. However, these genes do not appear to play a significant role in the effects of CBD in females.

### 4.1. The Effects of CBD on the Behavioral Phenotype in UCMS Rats

We found sex differences in the effects of UCMS on immobility in males and females, suggesting an opposite impact: UCMS increased passive coping in males but decreased it in females compared to a non-stressed control group. However, in both sexes, the administration of CBD restored this phenotype. Previous studies that examined the effects of UCMS on immobility in females showed contradictory results, some showing increased or decreased immobility, others showing no effect, depending on the stress protocol, the rodent type, etc. [45,46,47]. In our study, we saw lower baseline immobility measures in females compared to males, which were similar to those that were found in another study that showed lower immobility in female rats due to chronic stress. In that study, Long–Evans female rats bred for low- and high-anxiety-like behavior also displayed reduced immobility following CMS compared to controls [47]. This suggests that females are less prone to immobility behavior to begin with, implying that the FST might not be ideal to detect depressive phenotype in female rodents. Interestingly, it has been suggested that UCMS should be considered as the first stress session and FST as the second stress session and that females previously subjected to chronic mild stress cope better by exhibiting increased active behavior in the second FST in comparison with males [48]. This suggests that the behavioral paradigms assessing the stress response (for example, the combination of stressful procedures) may affect this sex-dependent outcome and should be considered in studying the pathophysiology of stress-related depression.

Moreover, UCMS-exposed males exhibited reduced swimming behavior, an effect that was prevented by CBD treatment. In contrast, no effect on active coping behaviors (swimming or climbing) was observed in females. Overall, males demonstrated higher levels of immobility compared to females, irrespective of stress exposure. This finding may reflect inherently lower baseline activity levels in novel or stressful environments among male rats compared to females [49], potentially contributing to their increased immobility in the FST.

In the OFT, exposure to UCMS similarly affected locomotor behavior in males and females, and UCMS-induced increase in locomotion was not restored with CBD treatment. We and others have previously shown increased locomotion following chronic stress [7,50,51], and an earlier study showed a similar effect of UCMS in female mice [46], see also [46,52]. The lack of effect of CBD on UCMS-induced hyperlocomotion aligns with studies showing similar results in different behavioral and genetic models [7,53], suggesting that CBD does not lead to changes in locomotor behavior. In addition, locomotion in non-UCMS rats was significantly higher in females compared to males, indicating that female rats exhibit higher locomotion behavior than males in a novel environment. Importantly, UCMS-exposed male and female rats showed no difference in the time spent in the center of the open field. This finding suggests a potential alteration in their exploratory activity rather than a straightforward increase in anxiety levels.

### 4.2. The Effects of CBD on Neuroinflammation in UCMS Rats

Sex-dependent differences in neuroinflammatory markers were also observed following exposure to UCMS and treatment with CBD. In males, CBD prevented the upregulation of the TNF-α gene in the CA1 and VS, suggesting a role of neuroinflammatory genes in the therapeutic-like effects of CBD in males exposed to UCMS. Positive correlations were observed between immobility and the neuroinflammatory genes, suggesting that their upregulation is highly associated with increased passive coping. These findings corroborate previous studies that showed a positive correlation between depressive symptoms and TNF-α and NF-κB1 expression, both in humans and animals, and specifically in the hippocampus [2,3,4,5,54,55,56,57,58]. CBD’s anti-inflammatory effect (i.e., decreasing the expression of TNF-α and NF-κB1) is also in line with other studies, which suggests that its antidepressant properties may be mediated by changes in these inflammatory markers in the hippocampus [14,59,60,61,62,63,64,65,66,67,68,69,70,71,72].

In both males and females, UCMS upregulated the NF-κB1 gene in the VS, with no effect from CBD. This effect was positively correlated with hyperactivity in the OFT, suggesting that hippocampal NF-κB1 may be involved in activity, in line with previous findings of lower locomotion in the OFT in *nfkb1*-knockout mice [73,74]. Baseline differences in the hippocampal expression of NF-κB1 between males and females could potentially explain the observed variation in locomotion. However, since gene expression was measured as fold change relative to baseline levels, we cannot confirm whether this disparity in NF-κB1 expression accounts for the locomotor differences, as baseline data for direct comparison was not included in the analysis. The observed differences in locomotor activity between female and male control rats could also be attributed to hormonal fluctuations, particularly due to the estrous cycle in females, which may influence their activity levels.

### 4.3. The Effects of CBD on CB1 and CB2 Genes in UCMS Rats

In males, but not in females, UCMS induced the downregulation of the CB1 gene in the VS that was prevented by CBD. The effect of UCMS corroborates with a previous study that demonstrated the downregulation of CB1 expression in the ventral hippocampus of male rats—but not females—exposed to the chronic mild stress (CMS) model of depression [75]. Notably, following CMS, these rats demonstrated anhedonic behavior. An earlier study showed similar results regarding the downregulation of hippocampal CB1 following UCMS [76]. Neither CBD nor UCMS alone significantly affected *cnr1* expression in the CA1 region. However, their combination resulted in a marked upregulation of CB1 receptor expression, indicating a synergistic effect between CBD and stress exposure. These differences in CBD’s effects across various brain regions, particularly within different hippocampal areas, highlight the complexity of the endocannabinoid system and the diverse mechanisms through which CBD exerts its actions.

UCMS males and females demonstrated the downregulation of the CB1 gene in the vmPFC, with no effect from CBD. This corroborates with findings from our previous study in UCMS males, showing the downregulation of *cnr1* in the vmPFC, with no effect of CBD [7]. Also, there was a negative correlation between this effect and the distance traveled in the OFT, suggesting that the downregulation is associated with increased locomotion. This is in line with previous findings regarding the involvement of CB1 in locomotion behavior [77,78,79].

Interestingly, CBD led to the downregulation of *cnr2* in the vmPFC in both sexes, irrespective of stress exposure. In males, CBD also induced the upregulation of *cnr2* in the CA1, but only in rats not exposed to stress. These effects were not correlated with behavioral measures, further emphasizing the broad and complex influence of CBD on endocannabinoid system mechanisms, including those not directly linked to depressive symptoms.

Evidence suggests a strong correlation between CB1 mRNA and protein levels in the context of stress and trauma, indicating that mRNA expression significantly influences protein regulation under certain pathological conditions. This relationship underscores the critical role of transcriptional activity in modulating receptor availability and function during physiological and disease states [80,81].

### 4.4. The Effects of CBD on Estrogen Genes in UCMS Rats

Sex-dependent differences were also observed in the effects of CBD on genes coding for estrogenic receptors following UCMS. In males, CBD prevented the UCMS-induced downregulation of VS ERα and ERβ genes. This downregulation of VS estrogenic receptors occurred concurrently with *tnf* elevation in the same region, in line with findings of TNF-α repressive properties of ERα and ERβ activation [82,83]. This association, however, was unique to the VS, implying that the neural mechanisms of interaction between the sex hormones and immune responses might be brain-region specific.

Negative correlations were observed between immobility in the FST and the estrogen genes, suggesting that their downregulation is highly associated with increased passive coping. It has been suggested that ERβ is involved in social and mood-related behaviors in males [84]. In fact, it was previously shown that male mice lacking the ERβ gene spent more time immobile and a reduced time swimming and climbing in the FST [84], further establishing a possible role of hippocampal ERβ in learned helplessness in males.

In UCMS-exposed rats of both sexes, the upregulation of CA1, *esr1*, and *esr2* was observed, with the latter positively correlated with the total distance traveled in the OFT. This suggests that increased CA1 ERβ levels are associated with increased locomotion. It has been shown that male ERβ knockout (BERKO) mice demonstrated deficits in motor behavior compared to control mice, thereby establishing a connection to ERβ [84].

Previous findings in female rodents showed that ERα and ERβ receptors are important mediators of the antidepressant effects of 17β-Estradiol and other ligands [39,40,41,42]. In our study, UCMS did not lead to the downregulation of hippocampal *esr1* in females. It is possible that the involvement of ERβ in mood-related behaviors in females is regulated through signaling in the central amygdala, as ERβ blocking in this region improved sucrose intake (i.e., an antidepressant effect) in female rats exposed to chronic stress [85]. Moreover, the central amygdala ERβ is involved in other behaviors, such as sociosexual behaviors and anxiety [86,87]. Notably, no differences were observed in the estrus cycle, suggesting that it is unlikely to account for the behavioral changes observed between the sexes in this study.

### 4.5. The Effects of CBD on miRNAs in UCMS Rats

In females, UCMS+CBD decreased CA1 miR-9-5p to control levels compared to the UCMS and CBD groups. This aligns with findings suggesting that MiR-9 is upregulated in the hippocampus of depressed mice and that silencing miR-9 in the hippocampus can improve depressive-like symptoms [21]. Yet, a different effect was observed in the VS in which UCMS exposure decreased the expression of VS miR-9-5p compared to the non-UCMS groups.

In UCMS-exposed females, CBD downregulated miR-98-5p in the VS compared to the control non-stressed groups, and this effect was negatively correlated with locomotion in the OFT, suggesting that miR-98-5p downregulation is associated with increased activity in females. In UCMS-exposed males, CBD upregulated miR-98-5p in the VS compared to the UCMS group; this aligns with a recent study suggesting that depressive symptoms are associated with lower levels of miR-98 in the hippocampus [22].

In UCMS-exposed females, CBD upregulated vmPFC miR-98-5p compared to the UCMS and CBD groups. This aligns with earlier studies showing male–female differences in miR-98 expression in different models [88,89].

In females, UCMS led to the downregulation of miR-146a-5p in the VS. In the vmPFC, CBD upregulated miR-146 in UCMS-exposed females compared to the UCMS group. These results are in line with several studies that showed that in both humans and animals, the elevation of miR-146 is associated with the worsening of depressive symptoms and vice versa [17,18,19,20].

In males, UCMS upregulated miR-146a-5p in the CA1 compared to the control groups, with no restoring effect of CBD, and this upregulation was positively correlated with increased immobility in the FST.

Overall, our results suggest that CBD modulates the expression of specific miRNAs in a region- and sex-specific manner in response to chronic stress, potentially contributing to its antidepressant effect.

## 5. Conclusions

This study aimed to investigate the molecular alterations in the brain associated with the therapeutic efficacy of CBD in male and female rats subjected to UCMS. Our findings reveal significant differences in behavioral responses between male and female rats under UCMS conditions, particularly in the FST. While CBD effectively mitigated UCMS-induced despair-like behavior in both sexes, it did not affect locomotor activity.

CBD effectively reversed UCMS-induced changes in immobility in both males and females. However, higher doses could yield differing effects, potentially enhancing therapeutic outcomes or revealing sex-specific responses. Specifically, higher doses can influence gene expression and physiological outcomes, which may vary by sex and depend on the specific experimental conditions.

Sex-specific variations emerged in the expression of neuroinflammatory markers and estrogen receptor genes. In males, CBD administration reversed UCMS-induced alterations in hippocampal CB1 expression, as well as inflammatory and estrogenic markers, suggesting the involvement of hippocampal cannabinoid, neuroinflammatory, and estrogenic mechanisms in CBD’s antidepressant-like effects. Conversely, CBD failed to reverse UCMS-induced changes in any markers or estrogenic receptors in females, indicating the potential engagement of alternative pathways.

The variety of effects of CBD observed in this study highlights the complexity of its mechanisms of action and the multiple pathways through which it affects stress and depression. While it is not yet fully understood whether the endocannabinoid, neuroinflammatory, and estrogenic changes induced by CBD are independent, there is compelling evidence suggesting that 17β-Estradiol can induce the overexpression of both CB1 and CB2 receptors [90,91], with CB2 also playing a role in anti-inflammatory processes [92]. Given that CBD affects both CB1 and CB2 receptors [15], it is likely that its effects across these pathways are interconnected rather than independent.

It is essential to recognize that male and female brains can respond in different manners to the same experimental manipulations. The lower immobility observed in females during the FST provides a different perspective on some of the findings presented here, emphasizing the need for cautious interpretation of certain molecular results in light of these behavioral differences.

Notably, there are indications of male–female differences in endocannabinoid, serotonergic, inflammatory, and estrogenic markers and activity, all of which may influence depressive symptoms [93,94,95,96,97,98,99,100,101,102,103,104,105,106,107]. The observed sex differences in stress response likely stem from a complex interplay of factors, including variations in serotonergic function (i.e., acting as an agonist at 5-HT1A receptors), fluctuations in the estrous cycle, and reducing the stress response within the hypothalamic–pituitary–adrenal (HPA) axis. The bidirectional crosstalk between sex hormones and stress hormones may shape the neurobiological and behavioral responses to UCMS and CBD treatment. For example, estrogen signaling could counteract the effects of stress-induced inflammation, as suggested by the CBD-mediated restoration of estrogen receptor expression in males. Also, stress hormones like glucocorticoids, which are elevated during UCMS, can exacerbate neuroinflammation and disrupt neuroendocrine balance [108,109]. This interplay can result in divergent effects on male and female brains, potentially explaining the observed sex differences in UCMS-induced immobility and gene expression patterns.

## Data Availability

The original contributions presented in this study are included in the article/Supplementary Material. Further inquiries can be directed to the corresponding author.

## Supplementary Materials

The following supporting information can be downloaded at: https://www.mdpi.com/article/10.3390/cells14020099/s1. Figure S1: A one-week example of UCMS schedule; Figure S2: Rat brain atlas illustrations indicating punch locations; Table S1: Rats’ weight (kg) during the experiment [Mean (SD)]; Table S2: rtPCR mRNA primer sequences; Table S3: Statistical analysis of behavioral tests and rtPCR results; Table S4: The distribution of estrus phases in each group of female rats was observed on the first day of behavioral tests; Table S5: Pearson’s correlation coefficients between estrus levels on the first day of the behavioral tests and the behavioral phenotype in female rats exposed to UCMS and CBD; Table S6: Pearson’s correlation coefficients between mRNA levels and the behavioral phenotype in male rats exposed to UCMS and CBD; Table S7: Pearson’s correlation coefficients between mRNA levels and the behavioral phenotype in female rats exposed to UCMS and CBD; Table S8: Pearson’s correlation coefficients between microRNA levels and the behavioral phenotype in male rats exposed to UCMS and CBD; Table S9: Pearson’s correlation coefficients between microRNA levels and the behavioral phenotype in female rats exposed to UCMS and CBD.
