# Supplementary material for: Cannabidiol Modulates Neuroinflammatory and Estrogen-Related Pathways in a Sex-Specific Manner in a Chronic Stress Model of Depression
Source: Cells. 2025 Jan 10;14(2):99. doi: 10.3390/cells14020099 (PMC11763596; doi:10.3390/cells14020099)
Supplement: Supplementary file 1 [file cells-14-00099-s001.zip › cells-3348207-supplementary.pdf]

Supplementary Material

Materials and Methods

Subjects

Unpredictable chronic mild stress (UCMS) protocol: Stressors are administered during a one-week UCMS protocol. The different stressors are applied in a rotating sequence to prevent habituation over a period of 4-6 weeks. These stressors include:

Cage soiling with water: A total of 300mL of water is added to the sawdust bedding in the cage, where it remains for 21 hours. Group housing: Each UCMS rat is paired with another UCMS rat of the same experimental group for social interaction. Different pairings are made each week to introduce novelty. Water deprivation: Rats undergo 18 hours of water deprivation to induce thirst-related stress. Empty water bottle: Following the water deprivation period, rats are presented with an empty water bottle for 1 hour. Food deprivation: Rats experience 20 hours of food deprivation to induce hunger-related stress. Restricted food access: After the food deprivation period, rats are given access to a very small amount of food for 1 hour. Overnight Illumination: Rats are exposed to overnight daylight illumination for 13 hours, from 6 pm until 7 am, disrupting their natural light-dark cycle. Cage tilting to 45°: Cages are tilted to a 45-degree angle for 17 hours, creating a physical challenge for the rats. Physical restraint: Rats are placed under physical restraint for 1 hour, restricting their movement while still allowing them to breathe freely.

This comprehensive protocol ensures a diverse range of stressors are experienced by the rats, mimicking the unpredictable and chronic nature of stress encountered in depressive disorders.

	Sunday	Monday	Tuesday	Wednesday	Thursday	Friday	Saturday
Food deprivation	13:00 ►	9:00		13:00 ►	9:00		
Restricted food		9:00 - 10:00			9:00 - 10:00		
Water deprivation			16:00 ►	10:00		16:00 ►	10:00
Empty water bottle				10:00 - 11:00			
Cage tilt 45°		16:00 ►	9:00			16:00 ►	9:00
Overnight illumination	18:00 ►	7:00			18:00 ►	7:00	
Soiled cage					12:00 ►	9:00	
Physical restraint	10:00-11:00					10:00-11:00	
Pair-housing				10:00 - 11:00			10:00 - 11:00

Figure S1: A one-week example of UCMS schedule.

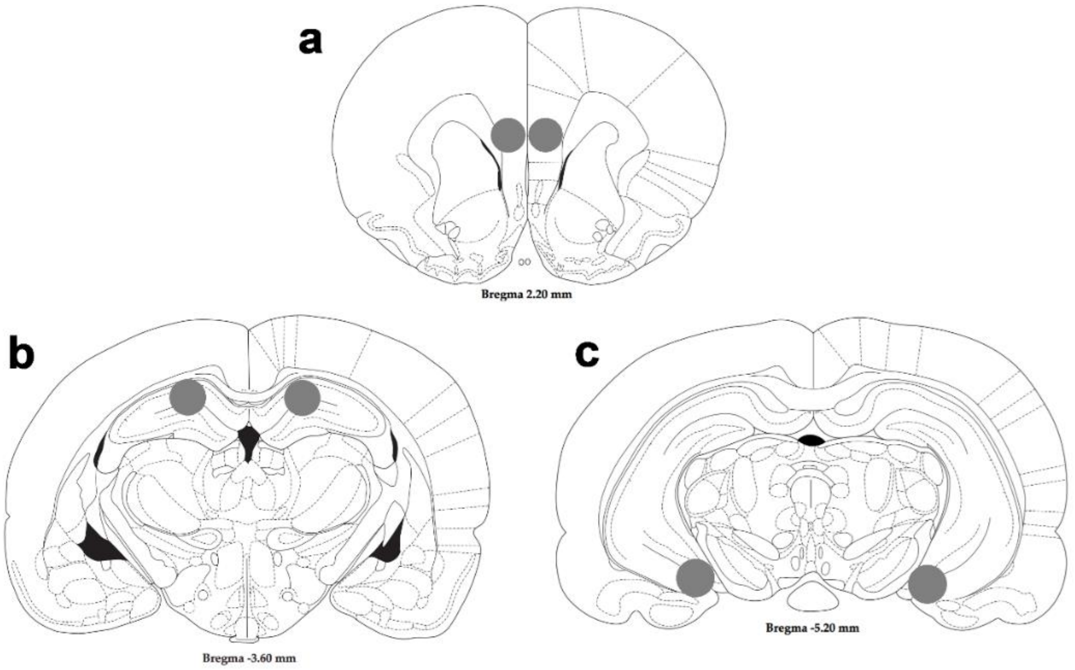

Figure S2 Rat brain atlas illustrations indicating punch locations. The numbers refer to the distance from Bregma. Gray circles indicate bilateral punches (1mm diameter) that were obtained from the (a) ventromedial prefrontal (vmPFC), (b) CA1, and (c) ventral subiculum (VS).

Results

Correlations between the expression of the different genes and behavior in males and females

Pearson bivariate correlations tests were conducted between the behavioral tests in males and females and the expression of ECS receptors (cnr1, cnr2), neuroinflammatory markers (tnf, nfkb1) and estrogen receptors (esr1, esr2) in the vmPFC, CA1, and VS. In males, for immobility in the FST, negative correlations were observed with cnr1 in the VS ($r=-0.397$, $p<0.05$), cnr2 in the CA1 ($r=-0.510$, $p<0.01$), VS esr1 ($r=-0.445$, $p<0.05$) and VS esr2 ($r=-0.436$, $p<0.05$), suggesting that downregulation of these genes was associated with increased passive coping. Positive correlations were observed between immobility and vmPFC tnf ($r=0.397$, $p<0.05$), CA1 tnf ($r=0.43$ $p<0.05$), VS tnf ($r=0.495$, $p<0.01$) and VS nfkb1 ($r=0.539$. $p<0.01$) suggesting that increased neuroinflammation was associated with increased immobility. For total distance in the OFT, a significant negative correlation was observed with the expression of vmPFC cnr1 ($r=-0.492$, $p<0.01$), suggesting that increased total distance was associated with the downregulation of the CB1r gene in the vmPFC. A positive correlation was seen with CA1 cnr1 ($r=0.468$, $p<0.01$), CA1 tnf ($r=0.336$, $p<0.05$), VS nfkb1 ($r=0.714$, $p<0.001$), and CA1 esr2 ($r=0.398$, $p<0.05$), suggesting that upregulation of these genes was associated with increased activity.

In females, for immobility in the FST, a negative correlation was observed with VS esr1 ($r=-0.353$, $p<0.05$), suggesting that upregulation of the ERα gene in the VS was associated with decreased immobility. For total distance in the OFT, a negative correlation was observed with vmPFC cnr1 ($r=-0.407$, $p<0.05$), suggesting increased activity was associated with the downregulation of the CB1r gene in the vmPFC. A positive correlation was seen with VS nfkb1 ($r=0.398$, $p<0.05$), CA1 esr2 ($r=0.440$, $p<0.01$), and VS esr2 ($r=0.479$, $p<0.01$), suggesting that upregulation of the NF-κB1 gene in the VS and the ERβ gene in the CA1 and VS was associated with increased activity.

Correlations between the expression of the microRNAs and behavior in males and females

Pearson bivariate correlation tests were conducted between the behavioral tests in males and females and the expression of miR-9-5p, miR-98-5p, and miR-146a-5p in the vmPFC, CA1, and VS. In males, for immobility in the FST, a positive correlation was observed with miR-146a-5p in the CA1 ($r=0.474$, $p<0.01$), suggesting that upregulation of this microRNA was associated with increased passive coping. For total distance in the OFT, a positive correlation was observed with miR-146a-5p in the CA1 ($r=0.428$, $p<0.05$), suggesting that upregulation of this microRNA was associated with increased activity.

In females, for immobility in the FST, positive correlations were observed with miR-98-5p ($r=0.346$, $p<0.05$) and miR-146a-5p in the CA1 ($r=0.392$, $p<0.05$), suggesting that upregulation of these microRNAs was associated with increased passive coping. For total distance in the OFT, a positive correlation was observed with miR-98-5p in the vmPFC ($r=0.519$, $p<0.01$), suggesting that upregulation of this microRNA was associated with increased activity. A negative correlation was observed with miR-98-5p in the VS ($r=-0.481$, $p<0.01$), suggesting that downregulation of this microRNA was associated with increased activity.

Table S1: Rats' weight (kg) during the experiment [Mean (SD)]

	Males				
	**Week 0**	**Week 1**	**Week 2**	**Week 3**	**Week 4**
**No UCMS - Veh**	177.4 (4.8)	225.4 (5.8)	273.4 (8.0)	308.6 (11.0)	335.2 (9.9)
**No UCMS - CBD**	181.0 (9.5)	232.0 (12.2)	283.0 (26.6)	321.0 (18.0)	352.2 (19.9)
**UCMS - Veh**	183.4 (6.8)	214.8 (10.3)	246.2 (15.2)	272.2 (22.3)	294.0 (10.7)
**UCMS - CBD**	177.8 (13.9)	204.4 (9.5)	231.0 (17.6)	268.0 (21.3)	287.4 (23.0)

	Females				
	**Week 0**	**Week 1**	**Week 2**	**Week 3**	**Week 4**
**No UCMS - Veh**	172.3 (6.4)	185.9 (6.2)	206.5 (7.3)	212.4 (7.8)	219.8 (8.8)
**No UCMS - CBD**	172.6 (6.6)	186.9 (6.0)	211.7 (10.2)	215.8 (11.3)	221.1 (9.9)
**UCMS - Veh**	178.8 (7.2)	185.2 (9.7)	200.0 (11.7)	208.6 (13.8)	214.3 (13.1)
**UCMS - CBD**	180.6 (8.1)	190.3 (7.5)	205.5 (7.9)	219.0 (9.3)	221.2 (9.9)

Table S2: rtPCR mRNA primer sequences

**mRNA**	**Protein Name**	**Forward primer**	**Reverse primer**
hprt1	HPRT	5′GAGCACTTCAGGGATTTGAATCA3′	5′GTAGATTCAACTTGCCGCTGCTGTCT3″
cnr1	CB1	5′CACCCATGGCTGAGGGTTC3′	5′CTGCAAGGCCATCTAGGATCGA3′
cnr2	CB2	5′GCCTGCAACTTCGTCATCTTC3′	5′TGCCGATCTTCAACAGGAA3′
tnf	TNF-α	5′CCAGACCCTCACACTCAGATC3′	5′CTCCGCTTGGTGGTTTGCTA3′
nfkb1	NF-κB1	5′GAGCTCCCCATCTTCAAC3′	5′ATTTGCCCAGTTCCGAAAGGATC3′
esr1	ERα	5’GGGAAGCTCCTGTTTGCTC3’	5’TGATTCCTGTCCAAG3’
esr2	ERβ	5'CCTCCTCAACTCCAGTATGT3’	5’GCTTCCGGCTACTTTCTGC3’

Table S3: Statistical analysis of behavioral tests and rtPCR results

Figure 1b	Number of Subjects (n)			Three-way ANOVA		Two-way ANOVA		
	Males	No UCMS - Veh	10	Sex	$F(1,79)=253.548$, $p<0.001$		Males	Females
**Immobility in the Forced Swim Test (FST)**		No UCMS - CBD	10	Drug	$F(1,79)=4.275$, $p<0.05$	Drug	$F(1,39)=19.412$, $p<0.001$	$F(1,39)=10.602$, $p<0.01$
		UCMS - Veh	10	Stress	$F(1,79)=15.833$, $p<0.001$	Stress	$F(1,39)=28.714$, $p<0<001$	$F(1,39)=1.314$, ns
		UCMS - CBD	10	Sex x Drug	$F(1,79)=29.777$, $p<0.001$	Stress x Drug	$F(1,39)=13.394$, $p<0.01$	$F(1,39)=7.199$, $p<0,05$
	Females	No UCMS - Veh	10	Sex x Stress	$F(1,79)=26.751$, $p<0.001$			
		No UCMS - CBD	10	Stress x Drug	$F(1,79)=2.988$, ns			
		UCMS - Veh	10	Sex x Stress x Drug	$F(1,79)=20.443$, $p<0.001$			
		UCMS - CBD	10					

Data not presented in the manuscript	Number of Subjects (n)			Three-way ANOVA		Two-way ANOVA		
	Males	No UCMS - Veh	10	Sex	$F(1,79)=18.23$, $p<0.001$		Males	Females
**Swimming in the Forced Swim Test (FST)**		No UCMS - CBD	10	Drug	$F(1,79)=2.155$, ns	Drug	$F(1,39)=19.412$, $p<0.001$	$F(1,39)=10.602$, $p<0.01$
		UCMS - Veh	10	Stress	$F(1,79)=5.769$, $p<0.05$	Stress	$F(1,39)=28.714$, $p<0<001$	$F(1,39)=1.314$, ns
		UCMS - CBD	10	Sex x Drug	$F(1,79)=8.598$, $p<0.01$	Stress x Drug	$F(1,39)=13.394$, $p<0.01$	$F(1,39)=7.199$, $p<0,05$
	Females	No UCMS - Veh	10	Sex x Stress	$F(1,79)=3.444$, ns			
		No UCMS - CBD	10	Stress x Drug	$F(1,79)=0.075$, ns			
		UCMS - Veh	10	Sex x Stress x Drug	$F(1,)=18.23$, $p<0.001$			
		UCMS - CBD	10					

Data not presented in the manuscript	Number of Subjects (n)			Three-way ANOVA		Two-way ANOVA		
	Males	No UCMS - Veh	10	Sex	$F(1,79)=38.766$, $p<0.001$		Males	Females

**Climbing in the Forced Swim Test (FST)**		No UCMS - CBD	10	Drug	F(1,79)=0.105, ns	Drug	F(1,39)=0, ns	F(1,39)=0.938, ns
		UCMS - Veh	10	Stress	F(1,79)=0.01, ns	Stress	F(1,39)=2.096, ns	F(1,39)=0.131, ns
		UCMS - CBD	10	Sex x Drug	F(1,79)=0.098, ns	Stress x Drug	F(1,39)=1.426, ns	F(1,39)=6.991, p<0.05
	Females	No UCMS - Veh	10	Sex x Stress	F(1,79)=2.077, ns			
		No UCMS - CBD	10	Stress x Drug	F(1,79)=0.832, ns			
		UCMS - Veh	10	Sex x Stress x Drug	F(1,)=4.08, p<0.05			
		UCMS - CBD	10					

**Figure 1c**	**Number of Subjects (n)**			**Three-way ANOVA**		**Two-way ANOVA**		
	Males	No UCMS - Veh	10	Sex	F(1,79)=114.64, p<0.001		Males	Females
**Total Distance in the Open Field Test (FST)**		No UCMS - CBD	10	Drug	F(1,79)=0.48, ns	Drug	F(1,39)=0, ns	F(1,39)=0.129, ns
		UCMS - Veh	10	Stress	F(1,79)=247.715, p<0.001	Stress	F(1,39)=2.096, ns	F(1,39)=0.755, ns
		UCMS - CBD	10	Sex x Drug	F(1,79)=0.488, ns	Stress x Drug	F(1,39)=1.426, ns	F(1,39)=2.738, ns
	Females	No UCMS - Veh	10	Sex x Stress	F(1,79)=35.864, p<0.001			
		No UCMS - CBD	10	Stress x Drug	F(1,79)=0.006, ns			
		UCMS - Veh	10	Sex x Stress x Drug	F(1,)=12.519, p<0.001			
		UCMS - CBD	10					

**Data not presented in the manuscript**	**Number of Subjects (n)**			**Three-way ANOVA**		**Two-way ANOVA**		
	Males	No UCMS - Veh	10	Sex	F(1,79)=142.811, p<0.001		Males	Females
**Time in Center in the Open Field Test (FST)**		No UCMS - CBD	10	Drug	F(1,79)=0.297, ns	Drug	F(1,39)=0.239, ns	F(1,39)=0.061, ns
		UCMS - Veh	10	Stress	F(1,79)=3.064, ns	Stress	F(1,39)=2.081, ns	F(1,39)=0.988, ns
		UCMS - CBD	10	Sex x Drug	F(1,79)=0.08, ns	Stress x Drug	F(1,39)=3.847, ns	F(1,39)=0.929, ns
	Females	No UCMS - Veh	10	Sex x Stress	F(1,79)=0.481, ns			
		No UCMS - CBD	10	Stress x Drug	F(1,79)=7.447, p<0.01			
		UCMS - Veh	10	Sex x Stress x Drug	F(1,)=2.903, ns			

		UCMS - CBD	10					

Figure 2a	Number of Subjects (n)			Three-way ANOVA		Two-way ANOVA		
	Males	No UCMS - Veh	8	Sex	F(1,70)=72.6, p<0.001		Males	Females
**vmPFC cnr1**		No UCMS - CBD	9	Drug	F(1,70)=0.03, ns	Drug	F(1,35)=0.011, ns	F(1,34)=0.098, ns
		UCMS - Veh	10	Stress	F(1,70)=35.34, p<0.001	Stress	F(1,35)=17.396, p<0.001	F(1,34)=17.912, p<0.001
		UCMS - CBD	9	Sex x Drug	F(1,70)=0.09, ns	Stress x Drug	F(1,35)=0.072, ns	F(1,34)=0.019, ns
	Females	No UCMS - Veh	8	Sex x Stress	F(1,70)=0.12, ns			
		No UCMS - CBD	9	Stress x Drug	F(1,70)=0.08, ns			
		UCMS - Veh	8	Sex x Stress x Drug	F(1,70)=0.01, ns			
		UCMS - CBD	10					

Figure 2b	Number of Subjects (n)			Three-way ANOVA		Two-way ANOVA		
	Males	No UCMS - Veh	9	Sex	F(1,74)=30.822, p<0.001		Males	Females
**CA1 cnr1**		No UCMS - CBD	9	Drug	F(1,74)=6.123, p<0.05	Drug	F(1,36)=9.757, p<0.01	F(1,37)=0.493, ns
		UCMS - Veh	10	Stress	F(1,74)=6.499, p<0.05	Stress	F(1,36)=13.617, p<0.001	F(1,37)=0.124, ns
		UCMS - CBD	9	Sex x Drug	F(1,74)=1.889, ns	Stress x Drug	F(1,36)=0.019, ns	F(1,37)=0, ns
	Females	No UCMS - Veh	10	Sex x Stress	F(1,74)=3.991, p<0.05			
		No UCMS - CBD	9	Stress x Drug	F(1,74)=0.006, ns			
		UCMS - Veh	10	Sex x Stress x Drug	F(1,74)=0.008, ns			
		UCMS - CBD	9					

Figure 2c	Number of Subjects (n)			Three-way ANOVA		Two-way ANOVA		
	Males	No UCMS - Veh	8	Sex	F(1,59)=314.635, p<0.001		Males	Females
**VS cnr1**		No UCMS - CBD	8	Drug	F(1,59)=1.18, ns	Drug	F(1,30)=8.394, p<0.01	F(1,28)=0, ns
		UCMS - Veh	7	Stress	F(1,59)=0.13, ns	Stress	F(1,30)=2.708, ns	F(1,28)=0.066, ns
		UCMS - CBD	8	Sex x Drug	F(1,59)=1.096, ns	Stress x Drug	F(1,30)=6.915, p<0.05	F(1,28)=0, ns
	Females	No UCMS - Veh	7	Sex x Stress	F(1,59)=0.724, ns			

		No UCMS - CBD	8	Stress x Drug	F(1,59)=0.935, ns			
		UCMS - Veh	7	Sex x Stress x Drug	F(1,59)=0.939, ns			
		UCMS - CBD	7					

**Figure 2d**	**Number of Subjects (n)**			**Three-way ANOVA**		**Two-way ANOVA**		
	Males	No UCMS - Veh	8	Sex	F(1,67)=431.328, p<0.001		Males	Females
**vmPFC cnr2**		No UCMS - CBD	9	Drug	F(1,67)=13.643, p<0.001	Drug	F(1,34)=6.927, p<0.05	F(1,32)=6.706, p<0.05
		UCMS - Veh	9	Stress	F(1,67)=0.022, ns	Stress	F(1,34)=0.614, ns	F(1,32)=0.233, ns
		UCMS - CBD	9	Sex x Drug	F(1,67)=0.057, ns	Stress x Drug	F(1,34)=0.081, ns	F(1,32)=0.454, ns
	Females	No UCMS - Veh	8	Sex x Stress	F(1,67)=0.776, ns			
		No UCMS - CBD	8	Stress x Drug	F(1,67)=0.487, ns			
		UCMS - Veh	8	Sex x Stress x Drug	F(1,67)=0.106, ns			
		UCMS - CBD	9					

**Figure 2e**	**Number of Subjects (n)**			**Three-way ANOVA**		**Two-way ANOVA**		
	Males	No UCMS - Veh	8	Sex	F(1,67)=135.766, p<0.001		Males	Females
**CA1 cnr2**		No UCMS - CBD	8	Drug	F(1,67)=2.643, ns	Drug	F(1,32)=22.341, p<0.001	F(1,34)=0.011, ns
		UCMS - Veh	9	Stress	F(1,67)=0.781, ns	Stress	F(1,32)=5.439, p<0.05	F(1,34)=0.001, ns
		UCMS - CBD	8	Sex x Drug	F(1,67)=3.308, ns	Stress x Drug	F(1,32)=1.302, ns	F(1,34)=0.004, ns
	Females	No UCMS - Veh	9	Sex x Stress	F(1,67)=0.666, ns			
		No UCMS - CBD	9	Stress x Drug	F(1,67)=0.129, ns			
		UCMS - Veh	9	Sex x Stress x Drug	F(1,67)=0.223, ns			
		UCMS - CBD	8					

**Figure 2f**	**Number of Subjects (n)**			**Three-way ANOVA**		**Two-way ANOVA**		
	Males	No UCMS - Veh	8	Sex	F(1,60)=793.088, p<0.001		Males	Females
**VS cnr2**		No UCMS - CBD	8	Drug	F(1,60)=0.106, ns	Drug	F(1,30)=0.208, ns	F(1,29)=0.024, ns
		UCMS - Veh	7	Stress	F(1,60)=0.587, ns	Stress	F(1,30)=2.174, ns	F(1,29)=0.039, ns

		UCMS - CBD	8	Sex x Drug	F(1,60)=0.001, ns	Stress x Drug	F(1,30)=0.29, ns	F(1,29)=0.093, ns
	Females	No UCMS - Veh	7	Sex x Stress	F(1,60)=0.157, ns			
		No UCMS - CBD	9	Stress x Drug	F(1,60)=0.005, ns			
		UCMS - Veh	7	Sex x Stress x Drug	F(1,60)=0.247, ns			
		UCMS - CBD	7					

**Figure 3a**	**Number of Subjects (n)**			**Three-way ANOVA**		**Two-way ANOVA**		
	Males	No UCMS - Veh	8	Sex	F(1,70)=5.397, p<0.05		Males	Females
**vmPFC tnf**		No UCMS - CBD	9	Drug	F(1,70)=1.592, ns	Drug	F(1,36)=1.06, ns	F(1,33)=0.576, ns
		UCMS - Veh	10	Stress	F(1,70)=1.262, ns	Stress	F(1,36)=1.211, ns	F(1,33)=0.243, ns
		UCMS - CBD	10	Sex x Drug	F(1,70)=0.035, ns	Stress x Drug	F(1,36)=0.122, ns	F(1,33)=1.894, ns
	Females	No UCMS - Veh	8	Sex x Stress	F(1,70)=0.18, ns			
		No UCMS - CBD	8	Stress x Drug	F(1,70)=0.532, ns			
		UCMS - Veh	8	Sex x Stress x Drug	F(1,70)=1.491, ns			
		UCMS - CBD	10					

**Figure 3b**	**Number of Subjects (n)**			**Three-way ANOVA**		**Two-way ANOVA**		
	Males	No UCMS - Veh	9	Sex	F(1,73)=50.789, p<0.001		Males	Females
**CA1 tnf**		No UCMS - CBD	9	Drug	F(1,73)=3.465, ns	Drug	F(1,36)=6.488, p<0.05	F(1,36)=0.086, ns
		UCMS - Veh	10	Stress	F(1,73)=5.44, p<0.05	Stress	F(1,36)=5.668, p<0.05	F(1,36)=1.097, ns
		UCMS - CBD	9	Sex x Drug	F(1,73)=1.99, ns	Stress x Drug	F(1,36)=4.085, ns	F(1,36)=0.003, ns
	Females	No UCMS - Veh	10	Sex x Stress	F(1,73)=0.527, ns			
		No UCMS - CBD	8	Stress x Drug	F(1,73)=1.802, ns			
		UCMS - Veh	10	Sex x Stress x Drug	F(1,73)=1.573, ns			
		UCMS - CBD	9					

**Figure 3c**	**Number of Subjects (n)**			**Three-way ANOVA**		**Two-way ANOVA**		
	Males	No UCMS - Veh	8	Sex	F(1,58)=30.721, p<0.001		Males	Females

**VS tnf**		No UCMS - CBD	7	Drug	F(1,58)=0.666, ns	Drug	F(1,29)=4.592, p<0.05	F(1,28)=0.388, ns
		UCMS - Veh	7	Stress	F(1,58)=0.561, ns	Stress	F(1,29)=3.704, ns	F(1,28)=0.29, ns
		UCMS - CBD	8	Sex x Drug	F(1,58)=3.267, ns	Stress x Drug	F(1,29)=6.718, p<0.05	F(1,28)=0.462, ns
	Females	No UCMS - Veh	8	Sex x Stress	F(1,58)=2.583, ns			
		No UCMS - CBD	7	Stress x Drug	F(1,58)=1.093, ns			
		UCMS - Veh	7	Sex x Stress x Drug	F(1,58)=4.527, p<0.05			
		UCMS - CBD	7					

**Figure 3d**	**Number of Subjects (n)**			**Three-way ANOVA**		**Two-way ANOVA**		
							Males	Females
	Males	No UCMS - Veh	7	Sex	F(1,67)=328.259, p<0.001			
**vmPFC nfkb1**		No UCMS - CBD	8	Drug	F(1,67)=8.763, p<0.01	Drug	F(1,32)=0.138, ns	F(1,34)=14.913, p<0.001
		UCMS - Veh	10	Stress	F(1,67)=1.45, ns	Stress	F(1,32)=1.565, ns	F(1,34)=0.195, ns
		UCMS - CBD	8	Sex x Drug	F(1,67)=5.9, p<0.05	Stress x Drug	F(1,32)=1.264, ns	F(1,34)=0.157, ns
	Females	No UCMS - Veh	8	Sex x Stress	F(1,67)=0.345, ns			
		No UCMS - CBD	9	Stress x Drug	F(1,67)=0.279, ns			
		UCMS - Veh	8	Sex x Stress x Drug	F(1,67)=1.17, ns			
		UCMS - CBD	10					

**Figure 3e**	**Number of Subjects (n)**			**Three-way ANOVA**		**Two-way ANOVA**		
							Males	Females
	Males	No UCMS - Veh	9	Sex	F(1,72)=106.527, p<0.001			
**CA1 nfkb1**		No UCMS - CBD	9	Drug	F(1,72)=4.983, p<0.05	Drug	F(1,36)=4.735, p<0.05	F(1,35)=0.966, ns
		UCMS - Veh	10	Stress	F(1,72)=10.139, p<0.01	Stress	F(1,36)=4.865, p<0.05	F(1,35)=5.283, p<0.05
		UCMS - CBD	9	Sex x Drug	F(1,72)=0.709, ns	Stress x Drug	F(1,36)=3.419, ns	F(1,35)=3.953, ns
	Females	No UCMS - Veh	10	Sex x Stress	F(1,72)=0.005, ns			
		No UCMS - CBD	9	Stress x Drug	F(1,72)=7.36, p<0.01			
		UCMS - Veh	9	Sex x Stress x Drug	F(1,72)=0.01, ns			
		UCMS - CBD	8					

Figure 3f	Number of Subjects (n)			Three-way ANOVA		Two-way ANOVA		
	Males	No UCMS - Veh	8	Sex	F(1,59)=290.817, p<0.001		Males	Females
VS nfkb1		No UCMS - CBD	8	Drug	F(1,59)=0.619, ns	Drug	F(1,31)=0.43, ns	F(1,27)=0.214, ns
		UCMS - Veh	7	Stress	F(1,59)=56.878, p<0.001	Stress	F(1,31)=44.953, p<0.001	F(1,27)=15.462, p<0.001
		UCMS - CBD	9	Sex x Drug	F(1,59)=0.043, ns	Stress x Drug	F(1,31)=1.547, ns	F(1,27)=0, ns
	Females	No UCMS - Veh	7	Sex x Stress	F(1,59)=6.878, p<0.05			
		No UCMS - CBD	7	Stress x Drug	F(1,59)=0.875, ns			
		UCMS - Veh	7	Sex x Stress x Drug	F(1,59)=0.903, ns			
		UCMS - CBD	7					

Figure 4a	Number of Subjects (n)			Three-way ANOVA		Two-way ANOVA		
	Males	No UCMS - Veh	8	Sex	F(1,70)=0.398, ns		Males	Females
vmPFC esr1		No UCMS - CBD	8	Drug	F(1,70)=0.305, ns	Drug	F(1,35)=0.031, ns	F(1,34)=0.561, ns
		UCMS - Veh	10	Stress	F(1,70)=0.144, ns	Stress	F(1,35)=0.503, ns	F(1,34)=0.762, ns
		UCMS - CBD	10	Sex x Drug	F(1,70)=0.541, ns	Stress x Drug	F(1,35)=2.601, ns	F(1,34)=0, ns
	Females	No UCMS - Veh	8	Sex x Stress	F(1,70)=1.258, ns			
		No UCMS - CBD	9	Stress x Drug	F(1,70)=0.742, ns			
		UCMS - Veh	8	Sex x Stress x Drug	F(1,70)=0.68, ns			
		UCMS - CBD	10					

Figure 4b	Number of Subjects (n)			Three-way ANOVA		Two-way ANOVA		
	Males	No UCMS - Veh	9	Sex	F(1,72)=90.666, p<0.001		Males	Females
CA1 esr1		No UCMS - CBD	8	Drug	F(1,72)=0.889, ns	Drug	F(1,35)=0.903, ns	F(1,36)=0.27, ns
		UCMS - Veh	10	Stress	F(1,72)=9.255, p<0.01	Stress	F(1,35)=5.65, p<0.05	F(1,36)=4.476, p<0.05
		UCMS - CBD	9	Sex x Drug	F(1,72)=0.005, ns	Stress x Drug	F(1,35)=2.064, ns	F(1,36)=1.744, ns
	Females	No UCMS - Veh	10	Sex x Stress	F(1,72)=0.256, ns			
		No UCMS - CBD	8	Stress x Drug	F(1,72)=3.512, ns			

		UCMS - Veh	10	Sex x Stress x Drug	F(1,72)=0.116, ns			
		UCMS - CBD	9					

Figure 4c	Number of Subjects (n)			Three-way ANOVA		Two-way ANOVA		
	Males	No UCMS - Veh	8	Sex	F(1,63)=0.743, ns		Males	Females
VS esr1		No UCMS - CBD	8	Drug	F(1,63)=0.243, ns	Drug	F(1,31)=12.044, p<0.01	F(1,31)=10.732, p<0.01
		UCMS - Veh	7	Stress	F(1,63)=1.484, ns	Stress	F(1,31)=6.01, p<0.05	F(1,31)=0.962, ns
		UCMS - CBD	9	Sex x Drug	F(1,63)=22.728, p<0.001	Stress x Drug	F(1,31)=13.03, p<0.01	F(1,31)=0.275, ns
	Females	No UCMS - Veh	7	Sex x Stress	F(1,63)=6.238, p<0.05			
		No UCMS - CBD	8	Stress x Drug	F(1,63)=9.472, p<0.01			
		UCMS - Veh	9	Sex x Stress x Drug	F(1,63)=5.73, p<0.05			
		UCMS - CBD	8					

Figure 4d	Number of Subjects (n)			Three-way ANOVA		Two-way ANOVA		
	Males	No UCMS - Veh	8	Sex	F(1,70)=65.014, p<0.001		Males	Females
vmPFC esr2		No UCMS - CBD	8	Drug	F(1,70)=0.521, ns	Drug	F(1,35)=0.004, ns	F(1,34)=0.755, ns
		UCMS - Veh	10	Stress	F(1,70)=1.69, ns	Stress	F(1,35)=2.008, ns	F(1,34)=0.28, ns
		UCMS - CBD	10	Sex x Drug	F(1,70)=0.413, ns	Stress x Drug	F(1,35)=4.821, p<0.05	F(1,34)=0.088, ns
	Females	No UCMS - Veh	8	Sex x Stress	F(1,70)=0.22, ns			
		No UCMS - CBD	9	Stress x Drug	F(1,70)=1.294, ns			
		UCMS - Veh	8	Sex x Stress x Drug	F(1,70)=2.57, ns			
		UCMS - CBD	10					

Figure 4e	Number of Subjects (n)			Three-way ANOVA		Two-way ANOVA		
	Males	No UCMS - Veh	9	Sex	F(1,72)=669.95, p<0.001		Males	Females
CA1 esr2		No UCMS - CBD	8	Drug	F(1,72)=5.911, p<0.05	Drug	F(1,35)=1.351, ns	F(1,36)=6.483, p<0.05
		UCMS - Veh	10	Stress	F(1,72)=20.278, p<0.001	Stress	F(1,35)=6.485, p<0.05	F(1,36)=17.306, p<0.001
		UCMS - CBD	9	Sex x Drug	F(1,72)=0.277, ns	Stress x Drug	F(1,35)=0.023, ns	F(1,36)=0.611, ns

	Females	No UCMS - Veh	10	Sex x Stress	F(1,72)=0.108, ns			
		No UCMS - CBD	8	Stress x Drug	F(1,72)=0.109, ns			
		UCMS - Veh	10	Sex x Stress x Drug	F(1,72)=0.333, ns			
		UCMS - CBD	9					

**Figure 4f**	**Number of Subjects (n)**			**Three-way ANOVA**		**Two-way ANOVA**		
	Males	No UCMS - Veh	8	Sex	F(1,63)=226.547, p<0.001		Males	Females
**VS esr2**		No UCMS - CBD	8	Drug	F(1,63)=11.569, p<0.01	Drug	F(1,31)=17.549, p<0.001	F(1,31)=0.115, ns
		UCMS - Veh	7	Stress	F(1,63)=0.074, ns	Stress	F(1,31)=4.25, p<0.05	F(1,31)=3.955, ns
		UCMS - CBD	9	Sex x Drug	F(1,63)=8.767, p<0.01	Stress x Drug	F(1,31)=12.194, p<0.01	F(1,31)=1.687, ns
	Females	No UCMS - Veh	7	Sex x Stress	F(1,63)=8.176, p<0.01			
		No UCMS - CBD	8	Stress x Drug	F(1,63)=12.228, p<0.001			
		UCMS - Veh	9	Sex x Stress x Drug	F(1,63)=3.264, ns			
		UCMS - CBD	8					

**Figure 5a**	**Number of Subjects (n)**			**Three-way ANOVA**		**Two-way ANOVA**		
	Males	No UCMS - Veh	7	Sex	F(1,65)=473.736, p<0.001		Males	Females
**vmPFC miR-9-5p**		No UCMS - CBD	9	Drug	F(1,65)=0.365, ns	Drug	F(1,31)=0.068, ns	F(1,33)=0.335, ns
		UCMS - Veh	7	Stress	F(1,65)=0.283, ns	Stress	F(1,31)=0.388, ns	F(1,33)=1.564, ns
		UCMS - CBD	9	Sex x Drug	F(1,65)=0.069, ns	Stress x Drug	F(1,31)=0.136, ns	F(1,33)=0.071, ns
	Females	No UCMS - Veh	8	Sex x Stress	F(1,65)=1.807, ns			
		No UCMS - CBD	9	Stress x Drug	F(1,65)=0.194, ns			
		UCMS - Veh	8	Sex x Stress x Drug	F(1,65)=0.002, ns			
		UCMS - CBD	9					

**Figure 5b**	**Number of Subjects (n)**			**Three-way ANOVA**		**Two-way ANOVA**		
	Males	No UCMS - Veh	9	Sex	F(1,69)=2305.741, p<0.001		Males	Females
**CA1 miR-9-5p**		No UCMS - CBD	9	Drug	F(1,69)=3.771, ns	Drug	F(1,34)=5.753, p<0.05	F(1,34)=1.026, ns
		UCMS - Veh	9	Stress	F(1,69)=1.023, ns	Stress	F(1,34)=3.535, ns	F(1,34)=0.052, ns

		UCMS - CBD	8	Sex x Drug	F(1,69)=0.012, ns	Stress x Drug	F(1,34)=5.938, p<0.05	F(1,34)=17.087, p<0.001
	Females	No UCMS - Veh	9	Sex x Stress	F(1,69)=0.358, ns			
		No UCMS - CBD	9	Stress x Drug	F(1,69)=22.838, p<0.001			
		UCMS - Veh	8	Sex x Stress x Drug	F(1,69)=7.25, p<0.01			
		UCMS - CBD	9					

**Figure 5c**	**Number of Subjects (n)**			**Three-way ANOVA**		**Two-way ANOVA**		
	Males	No UCMS - Veh	7	Sex	F(1,63)=119.777, p<0.001		Males	Females
**VS miR-9-5p**		No UCMS - CBD	7	Drug	F(1,63)=2.247, ns	Drug	F(1,28)=1.137, ns	F(1,34)=1.045, ns
		UCMS - Veh	7	Stress	F(1,63)=8.199, p<0.01	Stress	F(1,28)=0.016, ns	F(1,34)=24.911, p<0.001
		UCMS - CBD	8	Sex x Drug	F(1,63)=0.138, ns	Stress x Drug	F(1,28)=0.091, ns	F(1,34)=5.588, p<0.05
	Females	No UCMS - Veh	8	Sex x Stress	F(1,63)=6.961, p<0.05			
		No UCMS - CBD	9	Stress x Drug	F(1,63)=2.456, ns			
		UCMS - Veh	10	Sex x Stress x Drug	F(1,63)=1.079, ns			
		UCMS - CBD	8					

**Figure 5d**	**Number of Subjects (n)**			**Three-way ANOVA**		**Two-way ANOVA**		
	Males	No UCMS - Veh	7	Sex	F(1,66)=1354.861, p<0.001		Males	Females
**vmPFC miR-98-5p**		No UCMS - CBD	9	Drug	F(1,66)=1.154, ns	Drug	F(1,32)=0.122, ns	F(1,33)=1.305, ns
		UCMS - Veh	8	Stress	F(1,66)=5.239, p<0.05	Stress	F(1,32)=0.48, ns	F(1,33)=6.154, p<0.05
		UCMS - CBD	9	Sex x Drug	F(1,66)=0.362, ns	Stress x Drug	F(1,32)=0.194, ns	F(1,33)=24.91, p<0.001
	Females	No UCMS - Veh	8	Sex x Stress	F(1,66)=1.824, ns			
		No UCMS - CBD	9	Stress x Drug	F(1,66)=15.675, p<0.001			
		UCMS - Veh	8	Sex x Stress x Drug	F(1,66)=11.312, p<0.01			
		UCMS - CBD	9					

**Figure 5e**	**Number of Subjects (n)**			**Three-way ANOVA**		**Two-way ANOVA**		
	Males	No UCMS - Veh	8	Sex	F(1,68)=3.499, ns		Males	Females
**CA1 miR-98-5p**		No UCMS - CBD	9	Drug	F(1,68)=0.364, ns	Drug	F(1,33)=0.064, ns	F(1,34)=0.344, ns

		UCMS - Veh	9	Stress	F(1,68)=0.211, ns	Stress	F(1,33)=0.281, ns	F(1,34)=1.203, ns
		UCMS - CBD	8	Sex x Drug	F(1,68)=0.071, ns	Stress x Drug	F(1,33)=0.096, ns	F(1,34)=6.102, p<0.05
	Females	No UCMS - Veh	9	Sex x Stress	F(1,68)=1.363, ns			
		No UCMS - CBD	9	Stress x Drug	F(1,68)=2.641, ns			
		UCMS - Veh	8	Sex x Stress x Drug	F(1,68)=4.157, p<0.05			
		UCMS - CBD	9					

**Figure 5f**	**Number of Subjects (n)**			**Three-way ANOVA**		**Two-way ANOVA**		
	Males	No UCMS - Veh	7	Sex	F(1,63)=2149.541, p<0.001		Males	Females
**VS miR-98-5p**		No UCMS - CBD	7	Drug	F(1,63)=1.229, ns	Drug	F(1,28)=5.955, p<0.05	F(1,34)=2.378, ns
		UCMS - Veh	7	Stress	F(1,63)=9.737, p<0.01	Stress	F(1,28)=0.587, ns	F(1,34)=17.302, p<0.001
		UCMS - CBD	8	Sex x Drug	F(1,63)=8.702, p<0.01	Stress x Drug	F(1,28)=3.296, ns	F(1,34)=1.097, ns
	Females	No UCMS - Veh	8	Sex x Stress	F(1,63)=3.408, ns			
		No UCMS - CBD	9	Stress x Drug	F(1,63)=0.782, ns			
		UCMS - Veh	10	Sex x Stress x Drug	F(1,63)=4.557, p<0.05			
		UCMS - CBD	8					

**Figure 5g**	**Number of Subjects (n)**			**Three-way ANOVA**		**Two-way ANOVA**		
	Males	No UCMS - Veh	7	Sex	F(1,65)=1022.643, p<0.001		Males	Females
**vmPFC miR-146a-5p**		No UCMS - CBD	9	Drug	F(1,65)=3.913, ns	Drug	F(1,31)=1.246, ns	F(1,33)=2.812, ns
		UCMS - Veh	7	Stress	F(1,65)=0.106, ns	Stress	F(1,31)=0.16, ns	F(1,33)=0.005, ns
		UCMS - CBD	9	Sex x Drug	F(1,65)=0.195, ns	Stress x Drug	F(1,31)=0.064, ns	F(1,33)=7.823, p<0.01
	Females	No UCMS - Veh	8	Sex x Stress	F(1,65)=0.051, ns			
		No UCMS - CBD	9	Stress x Drug	F(1,65)=3.4, ns			
		UCMS - Veh	8	Sex x Stress x Drug	F(1,65)=4.803, p<0.05			
		UCMS - CBD	9					

**Figure 5h**	**Number of Subjects (n)**			**Three-way ANOVA**		**Two-way ANOVA**		
	Males	No UCMS - Veh	9	Sex	F(1,69)=7765.89, p<0.001		Males	Females

**CA1 miR-146a-5p**		No UCMS - CBD	9	Drug	$F(1,69)=0.484$, ns	Drug	$F(1,34)=0.286$, ns	$F(1,34)=0.259$, ns
		UCMS - Veh	9	Stress	$F(1,69)=1.131$, ns	Stress	$F(1,34)=13.529$, $p<0.001$	$F(1,34)=0.465$, ns
		UCMS - CBD	8	Sex x Drug	$F(1,69)=0.044$, ns	Stress x Drug	$F(1,34)=0.017$, ns	$F(1,34)=0.028$, ns
	Females	No UCMS - Veh	9	Sex x Stress	$F(1,69)=5.193$, $p<0.05$			
		No UCMS - CBD	9	Stress x Drug	$F(1,69)=0.008$, ns			
		UCMS - Veh	8	Sex x Stress x Drug	$F(1,69)=0.043$, ns			
		UCMS - CBD	9					

**Figure 5i**	**Number of Subjects (n)**			**Three-way ANOVA**		**Two-way ANOVA**		
	Males	No UCMS - Veh	7	Sex	$F(1,63)=209.989$, $p<0.001$		Males	Females
**VS miR-146a-5p**		No UCMS - CBD	7	Drug	$F(1,63)=0.015$, ns	Drug	$F(1,28)=0$, ns	$F(1,34)=0.048$, ns
		UCMS - Veh	7	Stress	$F(1,63)=1.204$, ns	Stress	$F(1,28)=0.888$, ns	$F(1,34)=9.293$, $p<0.01$
		UCMS - CBD	8	Sex x Drug	$F(1,63)=0.021$, ns	Stress x Drug	$F(1,28)=0.527$, ns	$F(1,34)=2.262$, ns
	Females	No UCMS - Veh	8	Sex x Stress	$F(1,63)=6.938$, $p<0.05$			
		No UCMS - CBD	9	Stress x Drug	$F(1,63)=0.108$, ns			
		UCMS - Veh	10	Sex x Stress x Drug	$F(1,63)=2.287$, ns			
		UCMS - CBD	8					

Table S4: The Distribution of estrus phases in each group of female rats was observed on the first day of behavioral tests

	No UCMS – Veh	No UCMS – CBD	UCMS – Veh	UCMS - CBD
Diestrus	3	4	3	4
Proestrus	4	2	4	3
Estrus	3	4	3	3

Table S5: Pearson correlation coefficients between estrus levels on the first day of the behavioral tests and the behavioral phenotype in female rats exposed to UCMS and CBD

	FST - Immobility	OFT - Total Distance
Estrus Level	r=-0.091	r=.084
	p=.576	p=.607

Table S6: Pearson correlation coefficients between mRNA levels and the behavioral phenotype in male rats exposed to UCMS and CBD

		FST - Immobility	OFT - Total Distance
cnr1	vmPFC	r=-.220	**r=-.492****
		p=.196	**p=.002**
	CA1	r=.053	**r=.468****
		p=.754	**p=.003**
	VS	**r=-.397***	r=-.303
		**p=.027**	p=.098
cnr2	vmPFC	r=.107	r=-.149
		p=.541	p=.393
	CA1	**r=-.510****	r=-.209
		**p=.002**	p=.243
	VS	r=.339	r=.330
		p=.062	p=.070
tnf	vmPFC	**r=.397***	r=.193
		**p=.015**	p=.253
	CA1	**r=.403***	**r=.336***
		**p=.013**	**p=.042**
	VS	**r=.495****	r=.280
		**p=.005**	p=.134
nfkb1	vmPFC	r=.048	r=.123
		p=.792	p=.496
	CA1	r=-.178	r=.223
		p=.293	p=.185
	VS	**r=.539****	**r=.714****
		**p=.001**	**p=.000**
esr1	vmPFC	r=-.005	r=.196
		p=.976	p=.253
	CA1	r=.087	r=.287
		p=.615	p=.090
	VS	**r=-.445***	r=-.346
		**p=.011**	p=.052
esr2	vmPFC	r=.056	r=.147
		p=.744	p=.391
	CA1	r=.186	**r=.398**
		p=.279	**p=.016**
	VS	**r=-.436***	r=p.347
		**p=.013**	p=.052

FST-forced swim test; OFT-open field test; vmPFC: ventromedial prefrontal cortex; VS: ventral subiculum.

Table S7: Pearson correlation coefficients between mRNA levels and the behavioral phenotype in female rats exposed to UCMS and CBD

		FST - Immobility	OFT - Total Distance
cnr1	vmPFC	r=.160	**r=-.407***
		p=.359	**p=.015**
	CA1	r=.099	r=.009
		p=.554	p=.957
	VS	r=.064	r=.042
		p=.740	p=.827
cnr2	vmPFC	r=-.308	r=.130
		p=.082	p=.470
	CA1	r=.073	r=-.117
		p=.676	p=.503
	VS	r=.046	r=.256
		p=.811	p=.172
tnf	vmPFC	r=-.253	r=-.032
		p=.150	p=.858
	CA1	r=-.157	r=.071
		p=.352	p=.676
	VS	r=.073	r=-.044
		p=.707	p=.819
nfkb1	vmPFC	r=-.282	r=.051
		p=.101	p=.773
	CA1	r=.195	r=.265
		p=.255	p=.119
	VS	r=.045	**r=.398***
		p=.821	**p=.036**
esr1	vmPFC	r=-.292	r=.183
		p=.089	p=.292
	CA1	r=-.058	r=.297
		p=.733	p=.074
	VS	**r=-.353***	r=.205
		**p=.047**	p=.260
esr2	vmPFC	r=-.115	r=.113
		p=.512	p=.518
	CA1	r=.095	**r=.440****
		p=.575	**p=.006**
	VS	r=.099	**r=.479****
		p=.589	**p=.006**

FST-forced swim test; OFT-open field test; vmPFC: ventromedial prefrontal cortex; VS: ventral subiculum.

Table S8: Pearson correlation coefficients between microRNA levels and the behavioral phenotype in male rats exposed to UCMS and CBD

		FST - Immobility	OFT - Total Distance
miR-9-5p	vmPFC	r=-.042	r=-.003
		p=.821	p=.988
	CA1	r=.165	r=-.170
		p=.343	p=.330
	VS	r=-.179	r=-.033
		p=.352	p=.866
miR-98-5p	vmPFC	r=.153	r=.157
		p=.396	p=.384
	CA1	r=.028	r=-.019
		p=.874	p=.913
	VS	r=-.343	r=-.072
		p=.068	p=.712
miR-146a-5p	vmPFC	r=-.039	r=-240
		p=.832	p=.187
	CA1	**r=.474****	**r=.428***
		**p=.004**	**p=.010**
	VS	r=.185	r=.051
		p=.336	p=.793

Table S9: Pearson correlation coefficients between microRNA levels and the behavioral phenotype in female rats exposed to UCMS and CBD

		FST - Immobility	OFT - Total Distance
miR-9-5p	vmPFC	r=.144	r=.249
		p=.418	p=.155
	CA1	r=-.018	r=-.221
		p=.919	p=.202
	VS	r=.245	r=-.333
		p=.156	p=.051
miR-98-5p	vmPFC	r=.110	**r=.519****
		p=.535	**p=.002**
	CA1	**r=.346***	r=.295
		**p=.042**	p=.086
	VS	r=.016	**r=-.481****
		p=.927	**p=.003**

miR-146a-5p	vmPFC	r=.193	r=.252
		p=.273	p=.151
	CA1	**r=.392***	r=-.048
		**p=.020**	p=.786
	VS	r=.334	r=-.069
		p=.050	p=.694
